# Supplementary material for: Control of type III protein secretion using a minimal genetic system
Source: Nat Commun. 2017 May 9;8:14737. doi: 10.1038/ncomms14737 (PMC5436071; doi:10.1038/ncomms14737)
Supplement: Supplementary Information — Supplementary Figures, Supplementary Tables, Supplementary Notes, and Supplementary References [file ncomms14737-s1.pdf]

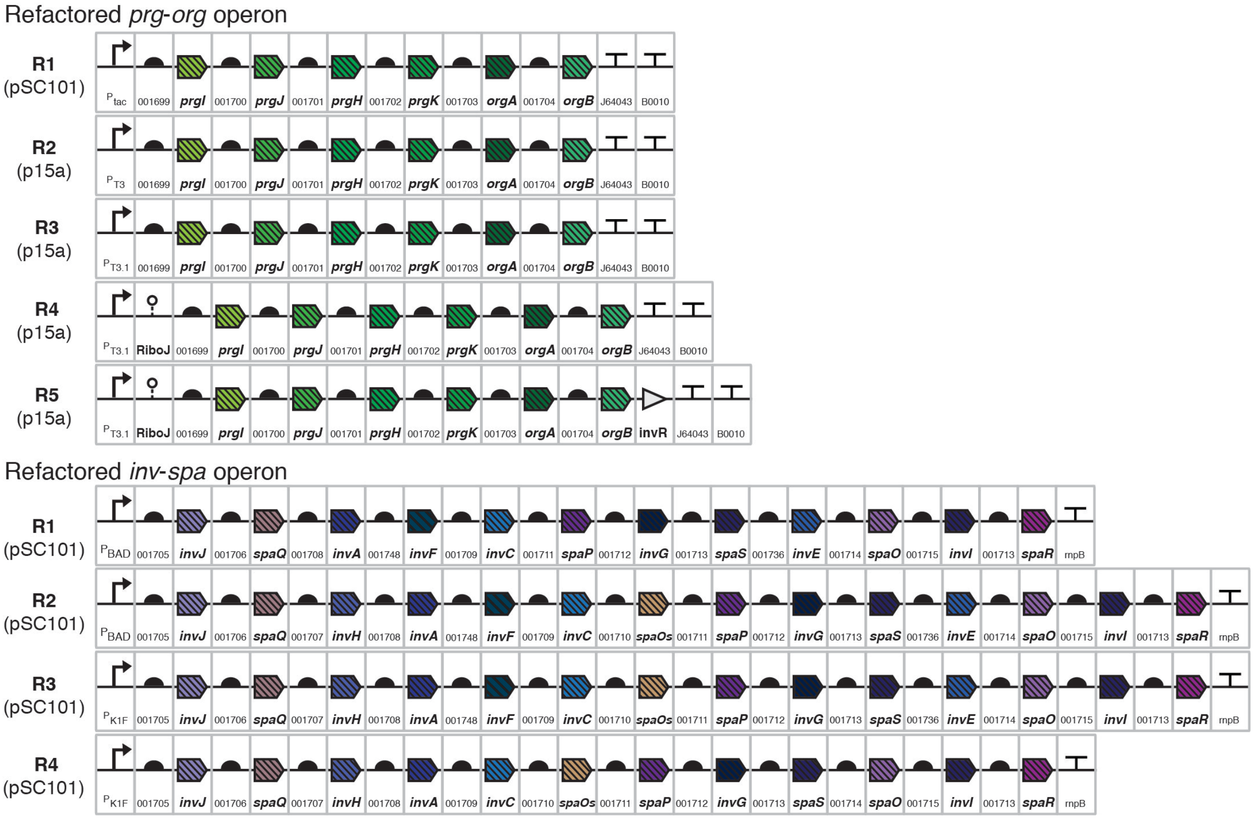

Supplementary Figure 1: **Variants of the refactored operons shown in Supplementary Table 1.** The maps of the plasmid backbones are shown in Supplementary Figure 18. Part sequences are provided in Supplementary Table 6. The final refactored operon was built by iterating over multiple steps. First, the individual operons were constructed and tested under inducible control in knockout strains where only that operon had been deleted from SPI-1. Next, the operons were combined and tested with the controller in a strain that contained both operons deleted, but the remainder of SPI-1 intact. Finally, the complete system was constructed and tested in a full knockout strain. During the second and third stages of this process, additional improvements were made to the original refactored operons. Flagella knockouts were made for structural analysis. Supplementary Table 2 and Supplementary Figure 1 show the variations of the system and plasmid version and strain genotype used for the experiments presented in the main text and SI figures.

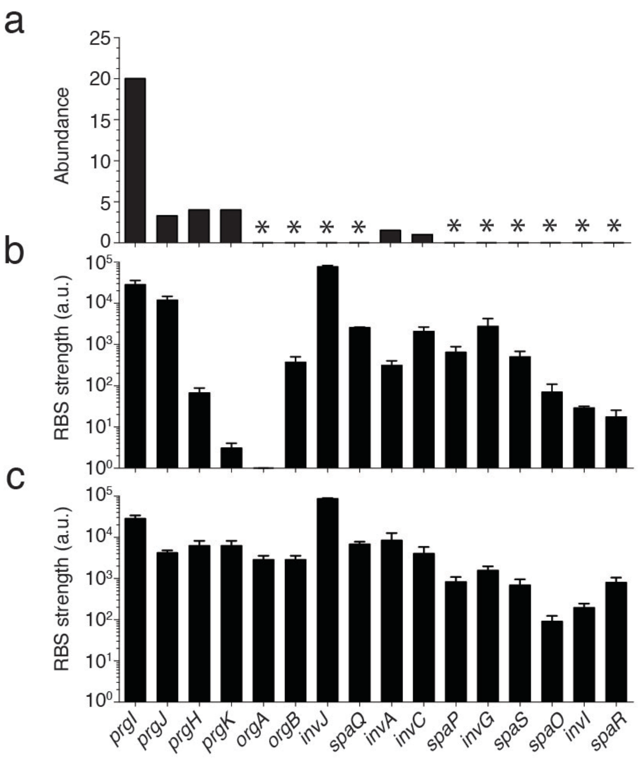

<u>Supplementary Figure 2:</u> **Data used to select synthetic RBSs in the construction of the first refactored operons. (a)** The molar ratios from previously published T3SS needle structures [1,2]. The *invC* gene was arbitrarily set to 1 and the abundances of other genes are relative to this value. Genes for which there was no information are marked with a star. **(b)** The strength of RBS genes measured for the native SPI-1 RBSs of each gene (Methods). The reporter system and measurements are described in the text and Methods. **(c)** The strength of the synthetic RBSs selected for each gene. The sequences are provided in Supplementary Table 6. The error bars represent the standard deviation of at least four replicates performed on different days.

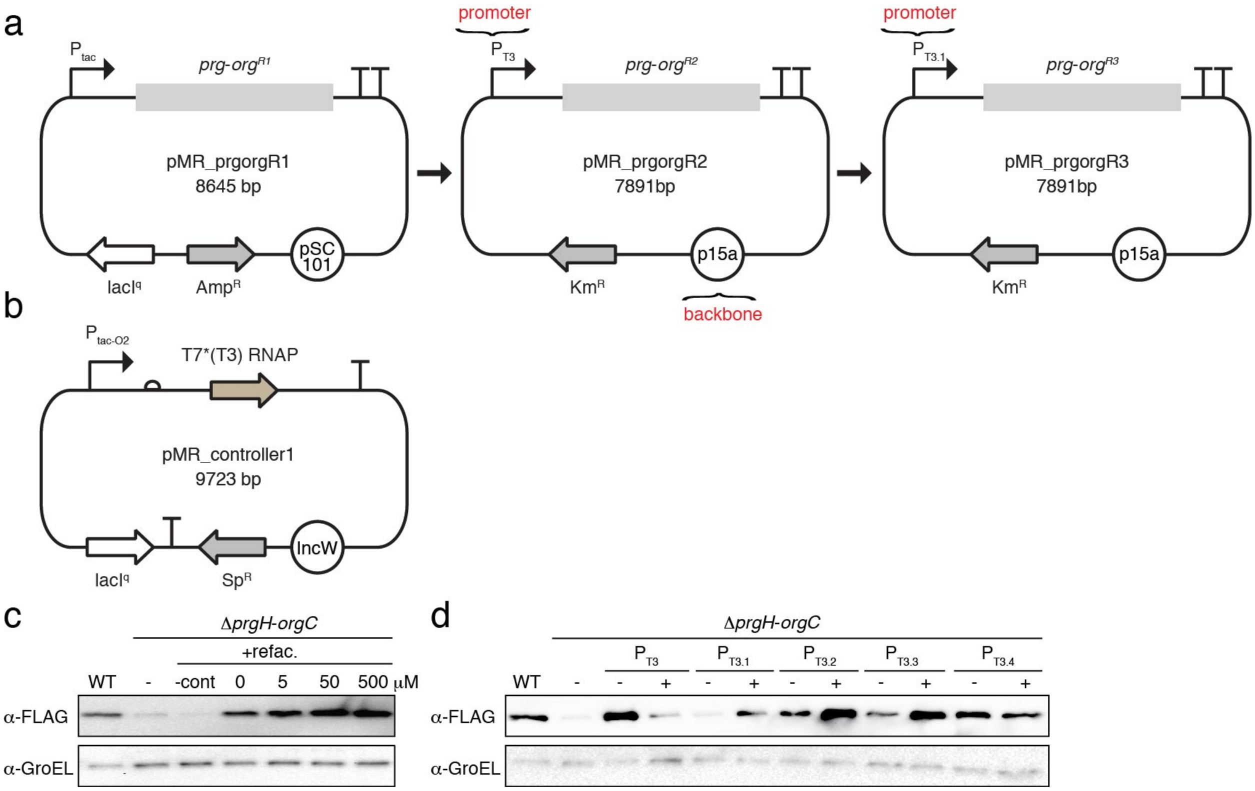

<u>Supplementary Figure 3:</u> Tuning of expression level of refactored *prg-org* operon. **(a)** Plasmids maps are shown for the refactored *prg-org* operons. The maps correspond to Supplementary Table 2 and Supplementary Figure 1. **(b)** The map of the controller plasmid for refactored *prg-org* operon is shown. T7*(T3) RNAP is under the control of $P_{tac\text{-}O2}$ promoter, which consists of 2 operator sites for $lacI^q$ (Supplementary Figure 17a). **(c)** Secretion assay of the refactored operon in Δ*prgH-orgC* (MR01) containing pMR_prgorgR2 (*prg-org*^{R2}) and pMR_controller1. Titration of inducer (IPTG; 0, 5, 50, 500 μM) was compared with the secretion from the Δ*prgH-orgC* strain containing only pMR_prgorgR2 (-cont). **(d)** A secretion assay is shown with *prg-org*^{R2} under the control of T3 promoters of increasing strength (left to right; [3]). Secretion was assayed in the Δ*prgH-orgC* strain (MR01). To test for inducibility, secretion was tested without (-) or with (+) 50 μM IPTG.

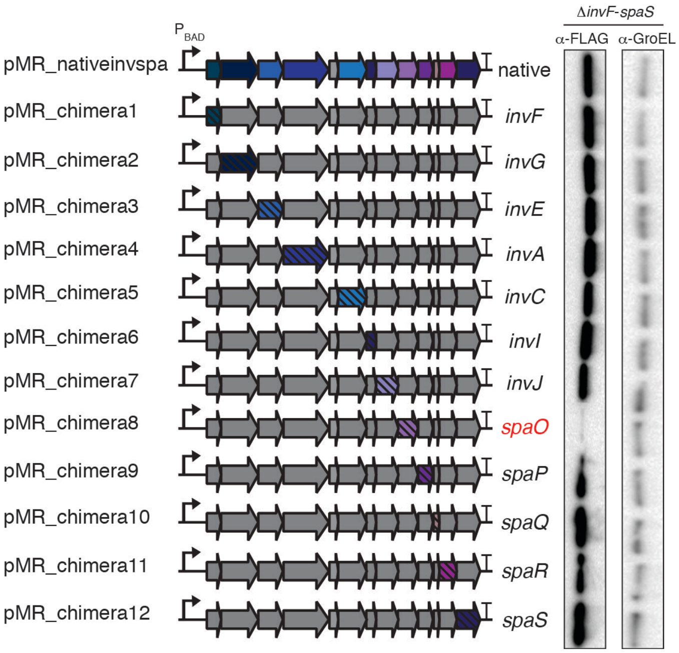

<u>Supplementary Figure 4:</u> Library of chimeric native/refactored *inv-spa* operons. Gibson assembly was used to swap the synthetic RBS and gene into each position of the native operon on the pMR_nativeinvspa plasmid. Secretion assays are shown when the operon is induced by 0.1 mM arabinose.

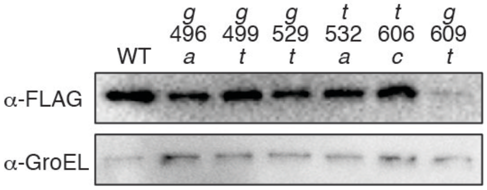

<u>Supplementary Figure 5:</u> Secretion assay for the *spaO* mutants in the context of the native *inv-spa* operon under P_{BAD} control. Arabinose was added at a concentration of 0.1 mM.

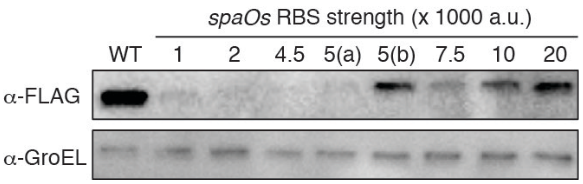

<u>Supplementary Figure 6:</u> The impact of the *spaOs* RBS strength on secretion is shown. The expression of refactored system was induced by 0.1 mM arabinose.

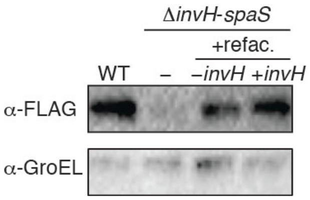

<u>Supplementary Figure 7:</u> Incorporation of *invH* increases secretion efficiency. Secretion efficiency was measured from the Δ*invH-spaS* strain (MR03) containing *inv-spa* operon with refactored *invH* under the 5000 au RBS control (*inv-spa*^{R2}). The expression of refactored operon was induced by 0.1 mM arabinose.

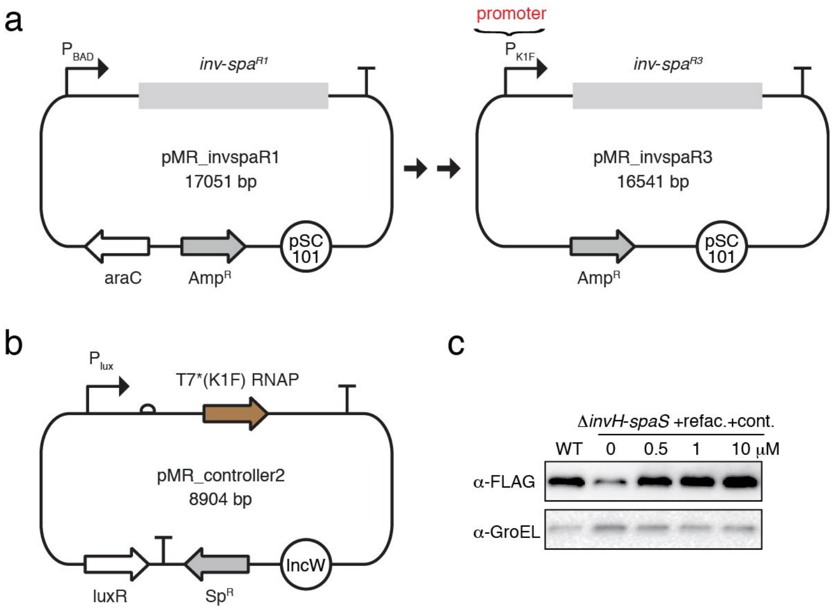

<u>Supplementary Figure 8:</u> Refactored *inv-spa* operon under the control of the T7*(K1F) RNAP. **(a)** Plasmid maps of refactored *inv-spa* operon are shown. **(b)** The plasmid map of the controller with T7*(K1F) RNAP is shown. **(c)** Secretion is analyzed from the Δ*invH-spaS* strain (MR03) containing *inv-spa*^{R3} and controller (pMR_controller2). The concentrations correspond to the titration of the inducer.

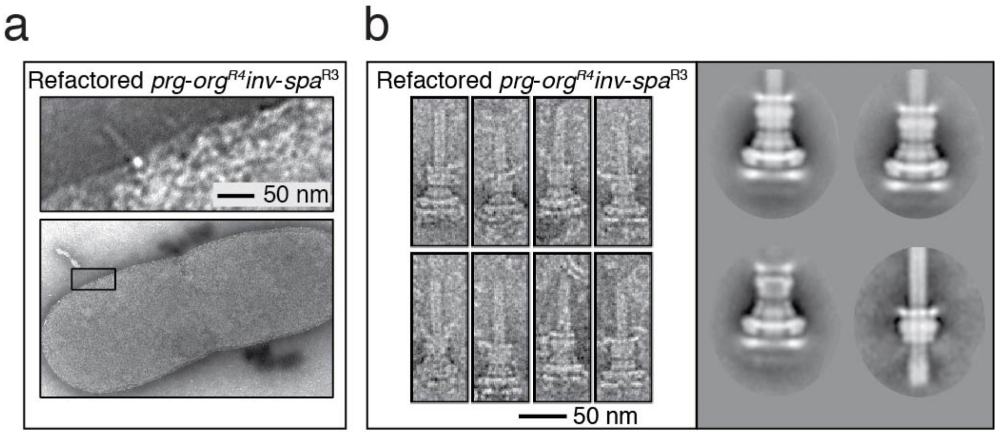

<u>Supplementary Figure 9:</u> **Structural analysis of the refactored *prg-org*^{R4}*inv-spa*^{R3} operons in the Δ*flhDC*Δ*prgH-orgC*Δ*invH-spaS* knockout (MR24).** The refactored operons were induced with 10 μM IPTG and 10 μM AHL. **(a)** EM analysis of the bacterial surface after osmotic shock as described [4]. **(b)** At left panels (both top and bottom), isolated needle complexes are shown. Particles from isolated needle complex are classified at right panel.

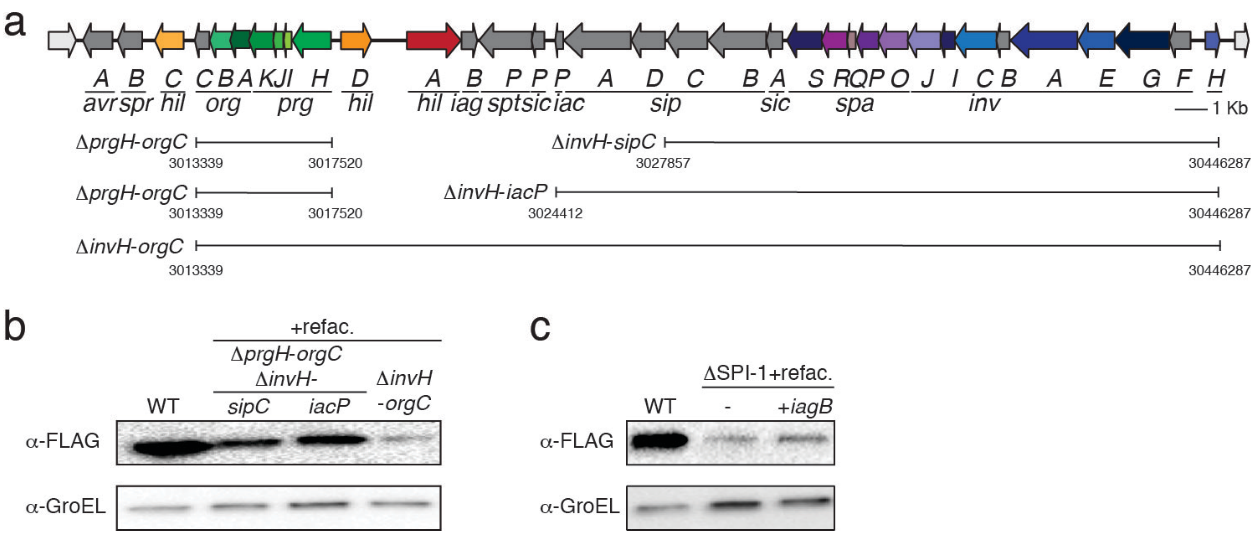

Supplementary Figure 10: Genetic screening for identification of additional requirements encoded within SPI-1. **(a)** A schematic is shown for additional deletions made to SPI-1. **(b)** A secretion assay was performed using the Δ*prgH-orgC*Δ*invH-sipC* or Δ*prgH-orgC*Δ*invH-iacP* or *ΔinvH-orgC* or strain containing the refactored system (*prg-org*^{R4}*inv-spa*^{R3}). **(c)** The *iagB* gene (native sequence) was introduced at the 3'-end of refactored *prg-org* operon. Assay was performed for functionality of refactored system without (lane 2) or with *iagB* (lane3).

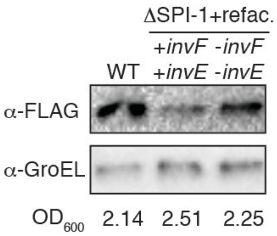

Supplementary Figure 11: **Secretion assay from refactored system in ΔSPI-1**. The SPI-1 knock out strain was complemented with refactored system containing invR on refactored *prg-org* operon together with (*prg-org*^{R5}*inv-spa*^{R3}) or without i*nvF*/*invE* (*prg-org*^{R5}*inv-spa*^{R4}) on *inv-spa* operon. The refactored operons were induced with 10 µM IPTG and 10 µM AHL.

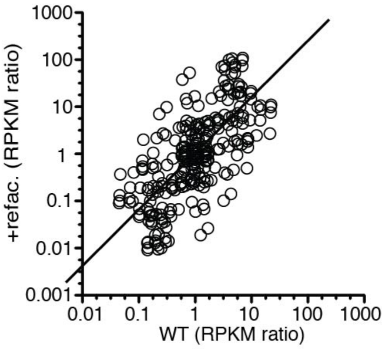

<u>Supplementary Figure 12:</u> **Regression of gene expression in the refactored system.** Median normalized RPKM values of the genes from wild type or the refactored SPI-1, fit to an x = y line (R^2 = 0.47). Data point corresponds to the expression ratios between all pairs of genes in the cluster; *prgI*, *prgJ*, *prgH*, *prgK*, *orgA*, *orgB*, *invR*, *invJ*, *spaQ*, *invH*, *invA*, *invC*, *spaP*, *invG*, *spaS*, *spaO*, *invI and spaR*. The average relative standard deviation (RSD) for the genes between biological replicates is 24 % (wild type) and 8 % (the refactored SPI-1).

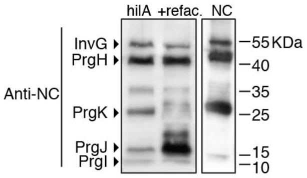

Supplementary Figure 13: **Expression level of needle complex proteins in strain SB905 (WT cluster, hilA overexpressed)[1] or the refactored system.** Needle complex components were analyzed by western blot analysis as a function of time. Each strain was induced by adding 0.012 % arabinose (w/v) or 10 μM IPTG/10 μM AHL, for SB905 or the refactored SPI-1, respectively when the media was changed from L-broth to inducing media (IM; LB containing 0.3 M NaCl (Methods). The needle complex was purified from strain SB905 at 6 hrs (NC) and used as a control.

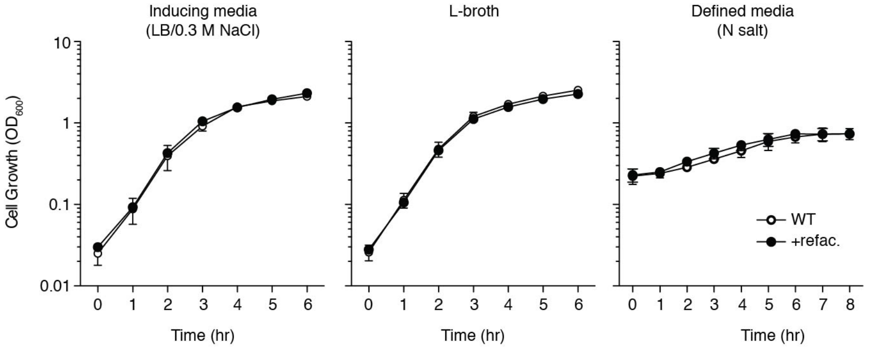

<u>Supplementary Figure 14:</u> Growth curves of strains containing refactored SPI-1 grown in different medias. The bacterial growth was measured from the strain expressing wild type or the refactored T3SS in inducing media (IM) or rich media without high osmolarity (L-broth) or defined media (N salt) (Figure 4a).The medias and growth experiments are described in the Methods. At each time point, samples were taken for measurement of optical density. For the refactored system, both inducers, IPTG and AHL, were added into each media as 10 μM, respectively.

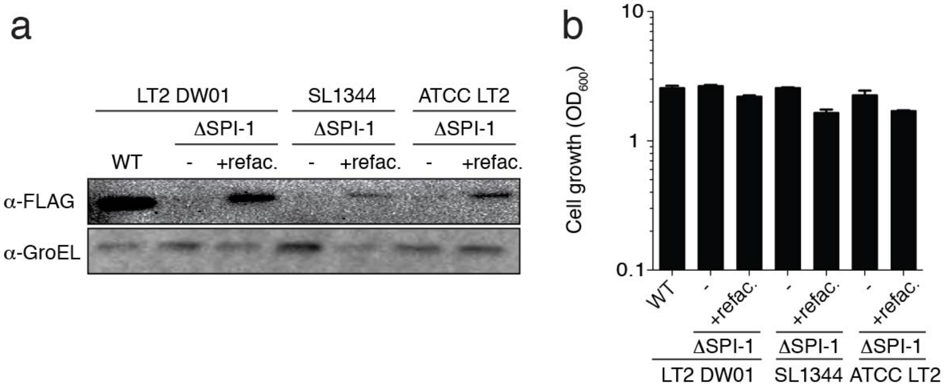

<u>Supplementary Figure 15:</u> Secretion assay using the refactored T3SS in different Salmonella strains. **(a)** Secretion assays are shown for the ΔSPI-1 mutants containing the refactored system (*prg-org*^{R5}*inv-spa*^{4}, pMR_controller3) with 10 μM AHL and 10 μM IPTG. The secretion assays were performed in L broth. The overnight cultures were freshly cultured in L-broth for 2 hrs at 250 rpm. The cultures were diluted 1:10 into 50 ml L-broth containing antibiotics and inducers if necessary and grown for 6 hrs. The secretion was compared to that from our wild type control (lane 1). **(b)** The cell density of each strain is shown at the same time that the secretion assay is performed.

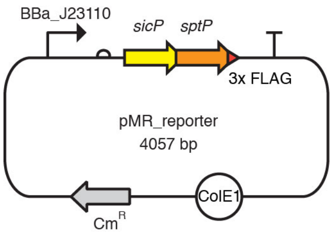

Supplementary Figure 16: **Reporter plasmid.** The SptP reporter is tagged with a 3x FLAG at C-terminus. The native *sicP*-*sptP* operon is preserved. The genes are expressed from the constitutive promoter, BBa_J23110 and native RBS of *sicP* (62 bp upstream of the ATG).

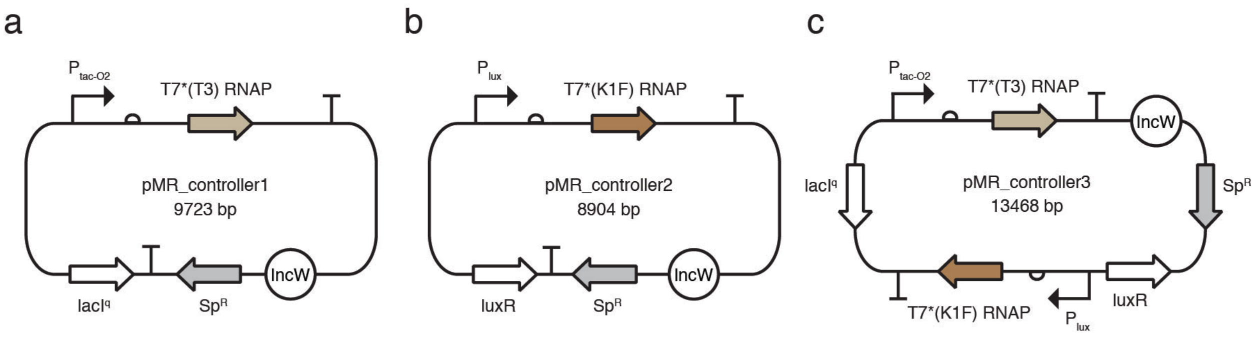

Supplementary Figure 17: **Controllers used in this study.** Two variants of T7 phage RNAP [T7*(T3) and T7*(K1F)] were used for controlling the refactored T3SS. The expression level of each RNAP was tuned by changing their RBS strength. This was done one at a time by designing a RBS library (AAGRAGSRGAAATGCWCS) for either T7*(T3) or T7*(K1F) and screening for those that produced >5-fold induction in *Salmonella* using mRFP and flow cytometery. **(a)** Map of T7*(T3) RNAP based controller for refactored *prg-org* cluster. To decrease expression of T7*(T3) under P_{tac} control, another *lacO* site was introduced ($P_{tac\text{-}O2}$). **(b)** Map of T7*(K1F) RNAP based controller for refactored *inv-spa* cluster. **(c)** Map of the unified controller with T7*(T3) and T7*(K1F).

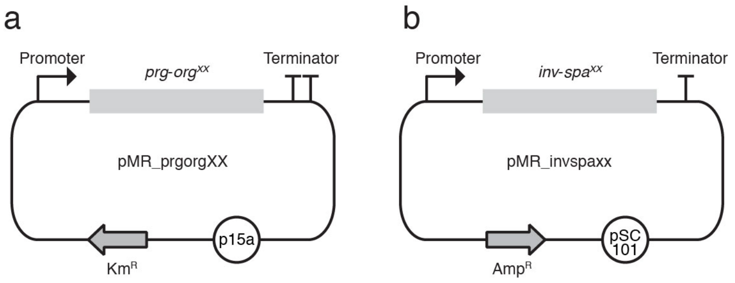

Supplementary Figure 18: Two backbone plasmids used for the refactored operon variants in Supplementary Figure 1. **(a)** Map of pMR_prgorgxx backbone plasmid. XX indicate each version of refactored plasmid (R1 to R5). Promoter indicates P_{tac}, P_{T3} and $P_{T3.1}$ from each version. Terminator indicates part registry terminators in order: BBa_J64043, BBa_B0010. The pSC101 origin of replication was replaced with p15a in pSB4A3. **(b)** Map of pMR_invspaxx backbone plasmid. XX indicate each version of refactored plasmid (R1 to R4). In each version, P_{BAD} or P_{K1F} was used as a promoter and *Salmonella rnpB* terminator was used on pSB4A3 backbone.

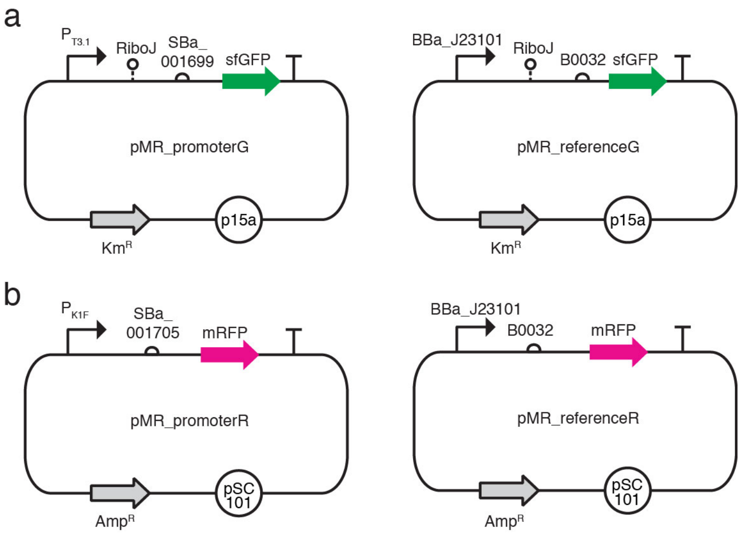

Supplementary Figure 19: **Reference standard for promoter activity normalization**. Construction of plasmids and measurements are described in Method. The fluorescent proteins, sfGFP or mRFP are under the control of constitutive promoter (BBa_J23101) followed by a 5' UTR (BBa_B0032) in each reference plasmid. **(a)** Map of pMR_promoterG for measurement of T3.1 promoter strength (left) and pMR_referenceG as a reference (right). **(b)** Map of pMR_promoterR for measurement of K1F promoter strength (left) and pMR_referenceR as a reference (right).

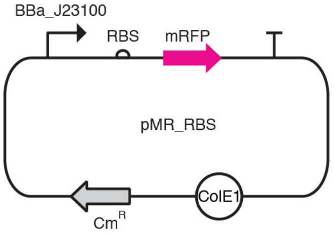

Supplementary Figure 20: **Plasmid map of the backbone used to generate the *Salmonella* RBS library.** Part registry RBSs (BBa_B0030, BBa_J61101, BBa_J61130) were introduced on ColE1 backbone plasmid, respectively. RBS library was built as described in Method. For measurement of RBS strength, mRFP was placed under the control of constitutive promoter, BBa_J23100.

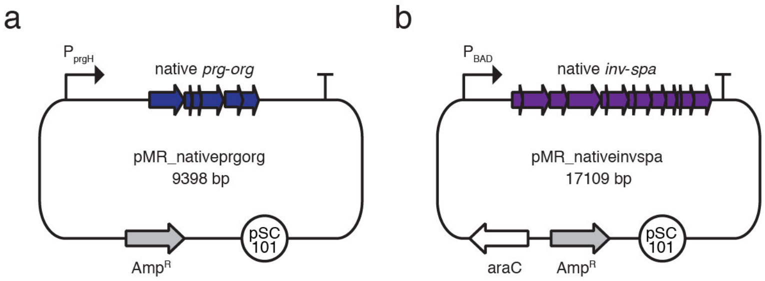

<u>**Supplementary Figure 21:**</u> **Plasmids used to test the complementation of the native *prg-org* (a) and *inv-spa* (b) operons on a plasmid backbone.** The native *prgH* promoter was cloned but the native *inv-spa* promoter could not be cloned into the backbone. Because of this, the P_{BAD} inducible promoter was used to induce the *inv-spa* operon. The genomic region from 3,013,185 to 3,017,809 was cloned for native *prg-org* and from 3,031,533 to 3,044,066 for native *inv-spa* (genomic region based on LT2: NC_003197.1).

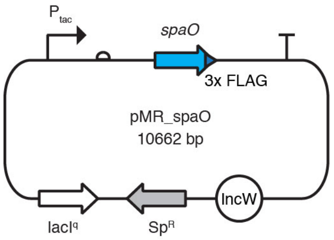

<u>Supplementary Figure 22:</u> **Plasmid map of 3x Flag-tagged native or synthetic *spaO* gene (Figure 2i).**

Figure 2a, 2d

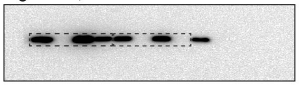

Figure 2c

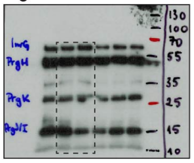

Figure 2e

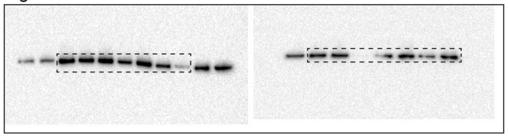

Figure 2f

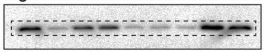

Figure 2h

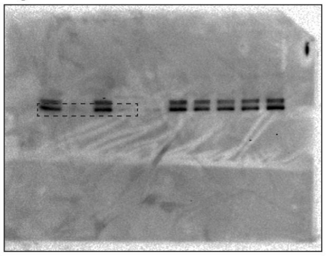

Figure 2i

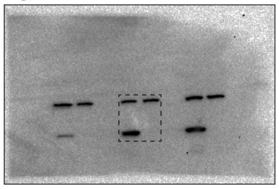

Figure 2j

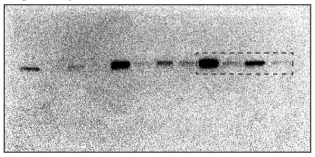

Figure 3b, α-FLAG

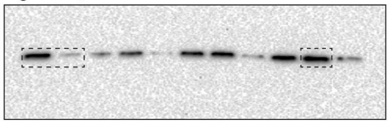

Figure 3b, α-GroEL

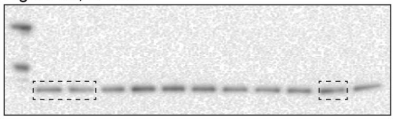

Figure 3c, α-FLAG

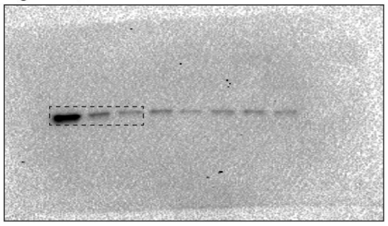

Figure 3c, α-GroEL

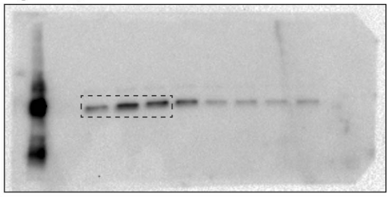

Figure 3e, α-FLAG

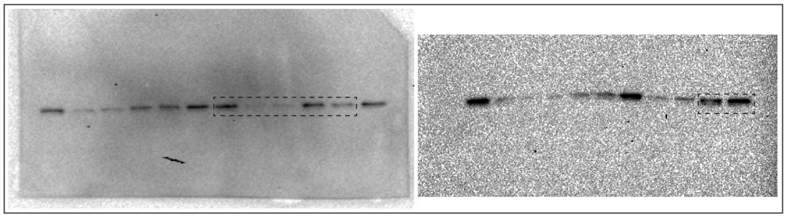

Figure 3e, α-GroEL

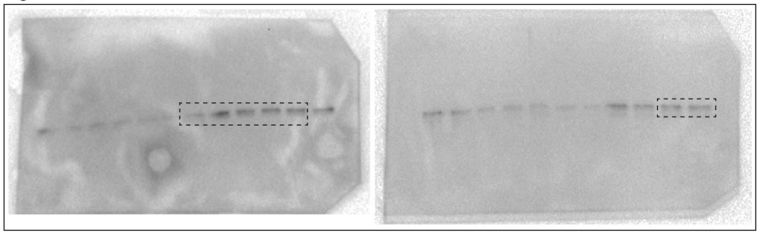

Figure 4a, α-FLAG

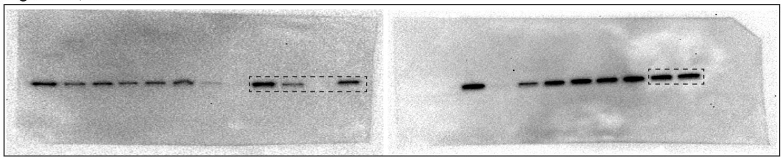

Figure 4a, α-GroEL

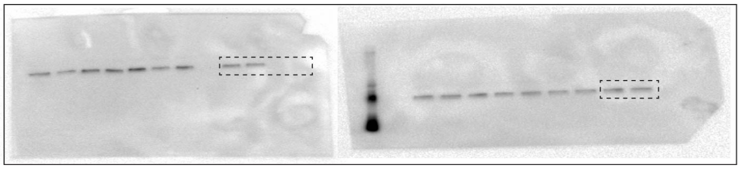

Supplementary Figure 23: Full images of western blot figures shown in the main paper.

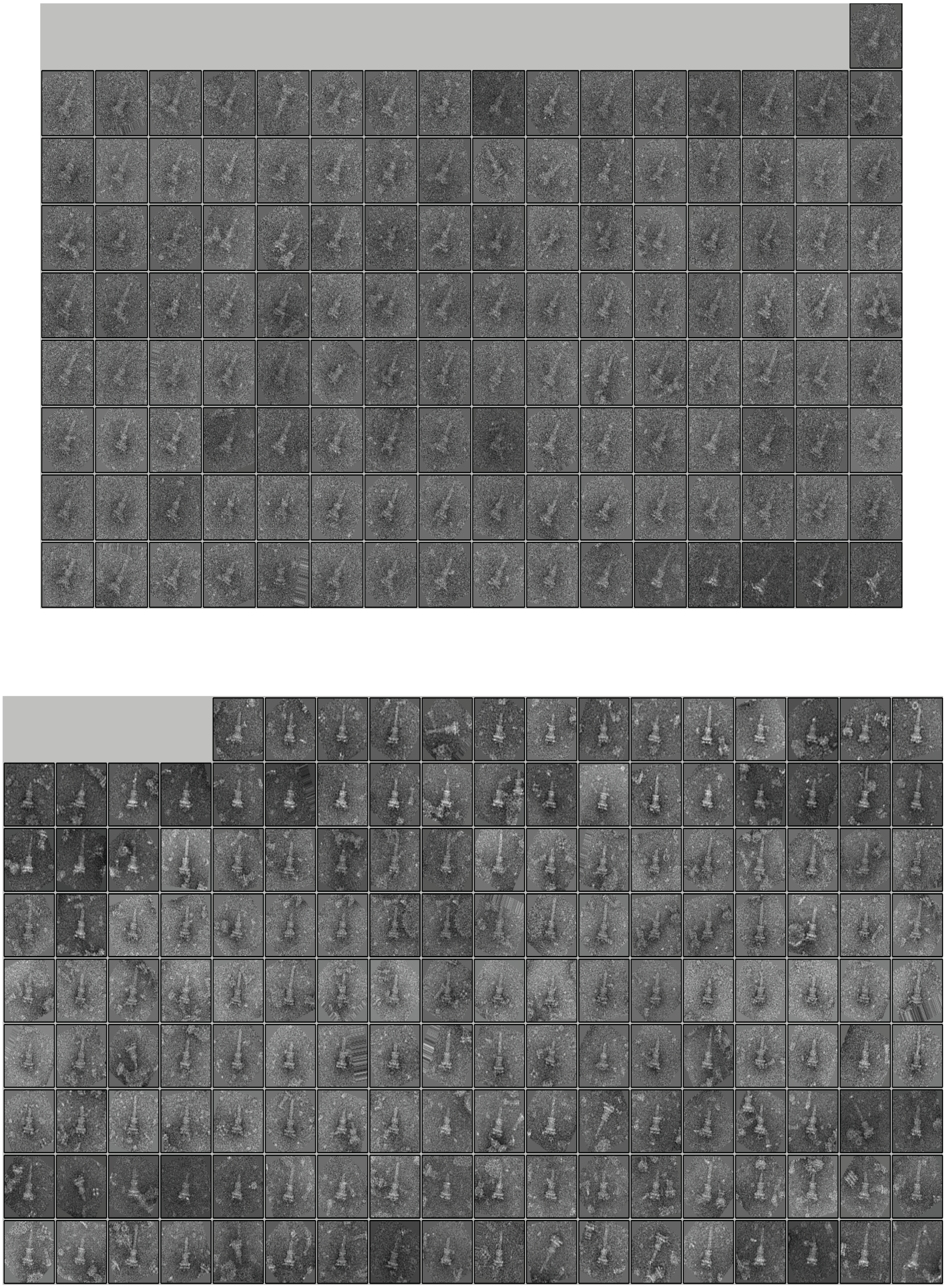

WT

REFACTORED

Supplementary Figure 24: The images of individual particles used for 2D-classification shown in Figure 4b (WT) and 4c (Refactored).

Supplementary Table 1: Genotype of Strain DW01

Gene	Genetic location[a,b]
aceF	180033~180034 (CG to GC)
yacH	184304~184305 (CG to GC)
rrsH	290716~290717 (C to A)
rrsH	290716~290717 (C to A)
brnQ	453937~453938 (C to T)
fimH	608855~608856 (G to C)
dcuC	691690~691691 (C to T)
STM0399	1016586~1016587 (CG to GC)
STM1043	1129916~1129931 (D)
yeaL	1357015~1357016 (I: G)
yeaG	1363417~1363418 (I: C)
mdtK	1503291~1503292 (CG to GC)
slyA	1520175~1520176 (CG to GC)
ydgJ	1536626~1536627 (CG to GC)
fumA	1543815~1543816 (I: C)
trg	1715972~1715973 (CG to GC)
rnb	1795045~1795046 (G to A)
hnr	1849647~1849648 (A to C)
yoaE	1926892~1926893 (I: GCG)
motA	2020199~2020200 (G to A)
wcaH	2191592~2191593 (I: C)
mglA	2286206~2286207 (A to T)
yeiG	2292158~2292159 (I: G)
nuoL	2427978~2427979 (C to G)
yfcY	2500653~2500654 (CG to GC)
yfeH	2537940~2537941 (CG to GC)
STM2438	2550664~2550665 (CG to GC)
STM2528	2661658~2661659 (G to C)
STM2589	2737590~2737591 (I: C)
STM2609	2757547~2757548 (I: G)
STM2632	2771659~2771660 (G to A), 2771862~2771863 (C to T), 2772072~2772073 (A to G), 2772288~2772289 (A to G), 2772485~2772486 (A to G), 2772535~2772536 (I: CTGAGACGCACCAGAACAACGATTCTGTGAGCCAATCTGAAC), 2772636~2772637 (A to G), 2772654~2772655 (T to C), 2772726~2772727 (A to G), 2773100~2773101 (T to C)
STM2633	2774323~2774324 (A to G)
hin	2914843~2914846 (D), 2914853~2914858 (D), 2914859~2914860 (A to T), 2914863~2914866 (D), 2914870~2914871 (AAC to CGT), 2914877~2914878 (I: CTT), 2914879~2914880 (I: AT), 2914880~2914881 (I: T), 2914883~2914884 (A to C), 2914886~2914887 (A to T), 2914888~2914889 (C to T), 2914891~2914892 (D), 2914895~2914896 (D), 2914897~2914901 (D), 2914903~2914904 (T to A), 2914906~2914907 (D), 2914910~2914911 (A to T), 2914912~2914913 (T to C), 2914913~2914914 (I: G), 2914917~2914918 (C to A), 2914919~2914920 (A to T), 2914921~2914922 (T to C), 2914926~2914927 (A to T), 2914927~2914928 (I: T), 2914928~2914929 (C to G), 2914930~2914931 (C to T), 2914931~2914932 (I: G), 2914932~2914933 (I: G), 2914939~2914940 (I: C), 2914941~2914942 (I: TG), 2914942~2914943 (C to T), 2914944~2914945 (I: TC), 2914948~2914949 (D), 2914950~2914951 (I: C), 2914954~2914955 (D), 2914956~2914957 (A to T), 2914958~2914959 (D), 2914960~2914961 (TG to AA), 2914964~2914965 (A to T), 2914966~2914967 (D), 2914967~2914968 (C to G), 2914971~2914972 (CCGG to TTAT), 2914976~2914977 (C to G), 2914980~2914983 (D), 2914984~2914985 (A to C), 2914990~2914991 (I: CACT), 2914992~2914993 (AG to TA), 2914999~2915000 (A to T)
hycC	2996777~2996778 (CG to GC)
hypD	3001699~3001700 (I: GCG)
sdaB	3123347~3123348 (CG to GC)
recC	3154018~3154019 (CG to GC)
ubiH	3219131~3219132 (CG to GC)
STM3091	3253331~3253332 (I: G)
yheU	3613714~3613715 (I: G)
pckA	3657649~3657650 (I: CGC)
malQ	3673616~3673617 (A to C)
malP	3674896~3674933 (D)
STM3633	3819802~3819803 (T to C)
hemE	4384226~4384227 (G to A)
rrsE	4396191~4396192 (D)
rpsR	4631399~4631400 (CG to GC)
STM4447	4689232~4689233 (I: G)
smp	4832866~4832867 (CG to GC)
Intergenic region	220823~220824(I: C), 288834~288835 (C to T), 288842~288843 (C to A), 288852~288853 (C to T), 510549~510550 (I:C), 520410~520411 (I:C), 1208417~1208418 (I: C), 1256604~1256605 (I: C), 1430154~1430155 (I: C), 1435558~1435559 (D), 1554185~1554186 (I: C), 1670336~1670337 (I: C), 1841403~1841404 (G to A), 2627554~2627555 (I: C), 2682820~2682821 (I: G), 2834544~2834545 (I:C), 2914778~2914779 (D), 2914781~2914782 (G to C), 2914786~2914787 (CC to GT), 2914789~2914791 (D), 2914793~2914794 (I: CC), 2914796~2914800 (D), 2914801~2914802 (I: T), 2914802~2914803 (G to T), 2914803~2914804 (I:C), 2914806~2914809 (D), 2914809~2914810 (A to G), 2914810~2914811 (I: G), 2914813~2914814 (T to A), 2914816~2914817 (I: AGG), 2914822~2914823 (D), 2914824~2914825 (I: CACT), 2914827~2914830 (D), 2914832~2914833 (I: C), 2914834~2914835 (G to T), 2914838~2914840 (D), 2914841~2914842 (A to T), 2915500~2915501 (I: T), 2915501~2915502 (I:T), 2915502~2915503 (A to G), 2915510~2915511 (D), 2915511~2915512 (AAA to TCC), 2915516~2915517 (I:C), 2915519~2915520 (A to C), 2915522~2915523 (A to G), 2915524~2915525 (T to G), 2915526~2915527 (D), 2915528~2915529 (A to T), 2915531~2915532 (TA to CC), 2915535~2915536 (A to T), 2915537~2915538 (CA to TT), 2915541~2915542 (T to C), 2915545~2915546 (G to C), 2915547~2915548 (G to C), 2915554~2915555 (G to T), 2915555~2915556 (I: GTT), 2915558~2915559 (T: GGC), 2915561~2915565 (D), 2915567~2915568 (C to G), 2915569~2915570 (TA to GG), 2915572~2915573 (D), 2915575~2915576 (A to G), 2915576~2915577 (I: A), 2915580~2915581 (CA to TT), 2915584~2915585 (T to C), 2915585~2915586 (I: C), 2915586~2915587 (I: G), 2915588~2915589 (G to T), 2915592~2915594 (D), 2915594~2915595 (A to G), 2915597~2915598 (D), 2915605~2915606 (D), 2915607~2915608 (D), 2915609~2915610 (D), 2915610~2915611 (C to G), 2915612~2915613 (A to G), 2915614~2915615 (A to T), 2915619~2915620 (G to A), 2915621~2915622 (A to T), 2915623~2915624 (T to G), 2915626~2915627 (D), 2915629~2915630 (G to A), 2915631~2915632 (A to T), 2915634~2915635 (I: C), 2915636~2915637 (I: A), 2915638~2915639 (I: ACGC), 2915639~2915640 (I: G), 2915641~2915642 (T to A), 2915646~2915647 (A to G), 2915648~2915649 (A to T), 2915651~2915652 (D), 2915654~2915655 (A to T), 2915656~2915658 (D), 2915660~2915663 (D), 2915668~2915669 (ACG to GTT), 2915674~2915675 (I: CCC), 2915677~2915678 (I: T), 2915678~2915679 (I: T), 2915679~2915680 (I: CCC), 2915686~2915687 (I: GCC), 2915687~2915688 (I: TG), 2915689~2915690 (G to T), 2915693~2915694 (A to C), 2915695~2915696 (D), 2915698~2915699 (I: TTA), 2915701~2915705 (D), 2915706~2915707 (I: A), 2915712~2915715 (D), 2915718~2915719 (T to A), 2915721~2915724 (D), 2915725~2915726 (GG to TT), 2915728~2915729 (A to T), 2915730~2915731 (I: T), 2915731~2915732 (I: G), 2915732~2915733 (I: TTT), 2915734~2915735 (GG to CT), 2915738~2915739 (I: GG), 2915740~2915741 (AC to GG), 2915745~2915746 (I: C), 2915746~2915747 (G to T), 3127648~3127649 (D), 3676852~3676853 (I: A), 3694174~3694175 (I: G), 4031932~4031933 (I: C), 4350854~4350855 (CA to GC), 4433282~4433283 (I: CG), 4459984~4459985 (I: C), 4606271~4606272 (D), 4673418~4673419 (I: T), 4675813~4675814 (I: C), pSLT28092~28098[c] (D)

a. *Genetic location is based on the nucleotide numbers of genomic sequence (NC_003197.1)[a] or of pSLT plasmid (NC_003277.1)[c] in LT2 strain*
b. *I, insertion; D, deletion; N to N, conversion*

Supplementary Table 2: Strains and variations of refactored operons that appear in this study

Name[a]	Refactored operon[b]		Regulation[c]	Genotype[d]			Figure[e,f]
	prg-org	*inv-spa*					
DW01				Wild type			
MR05				Δ*flhDC*			2b left, 4b
MR15	*native prg-org*		P_{prgH}	Δ*prgH-orgC*			2a (lane 3)
MR16		native inv-spa	P_{BAD}	Δ*invF-spaS*			2d (lane 3), 2e (lane 1), 2f (lane 1), 2h (lane 3)
MR17	*prg-org*^{R1}	-	P_{tac}	Δ*prgH-orgC*			2a (lane 4)
MR18	-	*inv-spa*^{R1}	P_{BAD}		Δ*invF-spaS*		2d (lane 4)
MR19	-	*inv-spa*^{R2}	P_{BAD}		Δ*invH-spaS*		2j (lane3), S7 (lane4)
MR20	*prg-org*^{R2}	-	pMR_controller1	Δ*prgH-orgC*			S3c
MR21	-	*inv-spa*^{R3}	pMR_controller2		Δ*invH-spaS*		S8c
MR22	*prg-org*^{R3}	-	pMR_controller1	Δ*prgH-orgC*			S3d
MR23	*prg-org*^{R3}	*inv-spa*^{R3}	pMR_controller3	Δ*prgH-orgC*	Δ*invH-spaS*	Δ*flhDC*	2b right, 2c (left lane)
MR24	*prg-org*^{R4}	*inv-spa*^{R3}	pMR_controller3	Δ*prgH-orgC*	Δ*invH-spaS*	Δ*flhDC*	2c (right lane), S9
MR25	*prg-org*^{R4}	*inv-spa*^{R3}	pMR_controller3	Δ*prgH-orgC*	Δ*invH-spaS*		3b
MR26	*prg-org*^{R4}	*inv-spa*^{R3}	pMR_controller3	ΔSPI-1			3c (lane 3), 3e (lane 3)
MR27	*prg-org*^{R5}	*inv-spa*^{R4}	pMR_controller3	ΔSPI-1			3e (lane 4), 4a, 4d, 4e, S11 (lane 3)
MR28	*prg-org*^{R5}	*inv-spa*^{R3}	pMR_controller3	ΔSPI-1			S11 (lane 2)
MR29	*prg-org*^{R4}	*inv-spa*^{R4}	pMR_controller3	ΔSPI-1		Δ*ompD*	3e (lane5)
MR30	*prg-org*^{R5}	*inv-spa*^{R4}	pMR_controller3	ΔSPI-1		Δ*flhDC*	4c

a. All strains are based on the DW01 parent. Except DW01 parent strain, all strains contain the reporter plasmid (pMR_reporter; Supplementary Figure 16).
b. The superscripts refer to the variant of the operon shown in Supplementary Figure 1.
c. Gene expression was regulated by inducible promoter or by bacteriophage RNAP based-controller system on a plasmid.
d. Oligonucleotides sequences used for construction of genomic knockout were described in Supplementary Table 3.
e. The main text figures where this strain is used are listed.
f. The supplementary information figures where this strain is used are listed.

Supplementary Table 3: Generation of knockout strains

Strain[a]	Genotype	Oligonucleotides[b]	Source[c]
MR01	Δ*prgH-orgC*	**F:**cctgtgcggtaatctgctgctatcgagaacgacagacatctgtaggctggagctgcttc<br>**R:**acaccataccttatcacaaatcgccagcaatggggggttcacatatgaatatcctcctta	
MR02	Δ*invF-spaS*	**F:**agcatggtttatacagacgtgttccgcgcaaaagctgcattgtaggctggagctgcttc<br>**R:**aaaaacgcccaatgaatacatcgctactgccttacgcggccatatgaatatcctcctta	
MR03	Δ*invH-spaS*	**F:**taattatatcatgatgagttcagccaacggtgatatggcctgtaggctggagctgcttc<br>**R:**aaaaacgcccaatgaatacatcgctactgccttacgcggccatatgaatatcctcctta	
MR04	Δ*prgH-orgC* Δ*invH-spaS*		P22
MR05	Δ*flhDC*		[5]
MR06	Δ*flhDC*Δ*prgH-orgC* Δ*invH-spaS*		P22
MR07	ΔSPI-1	**F:**taattatatcatgatgagttcagccaacggtgatatggcctgtaggctggagctgcttc<br>**R:**gctggaaggatttcctctggcaggcaaccttataatttcacatatgaatatcctcctta	
MR08	Δ*invH-orgC*	**F:**taattatatcatgatgagttcagccaacggtgatatggcctgtaggctggagctgcttc<br>**R:**acaccataccttatcacaaatcgccagcaatggggggttcacatatgaatatcctcctta	
MR09	Δ*invH-iacP*	**F:**taattatatcatgatgagttcagccaacggtgatatggcctgtaggctggagctgcttc<br>**R:**cagccatacctgaaccgggtactacatgtgtgatgattttcatatgaatatcctcctta	
MR10	Δ*prgH-orgC* Δ*invH-iacP*		P22
MR11	Δ*iagB*	**F:**tacttttagaactatctgaaagtaagctatttctgtataatgtaggctggagctgcttc<br>**R:**taaaatctgagagaggagatatgcattatttttttatcatcatatgaatatcctcctta	
MR12	Δ*ompD*	**F:**gttgaggaaacacgctaagaaaattataaggattattaaatgtaggctggagctgcttc<br>**R:**ttgattttgtaatcaacgaaggtggacatgtttttgcatatgaatatcctcctta	
MR13	ΔSPI-1Δ*ompD*		P22
MR14	Δ*flhDC*ΔSPI-1		P22
MR31	Δ*invH-sipC*	**F:**taattatatcatgatgagttcagccaacggtgatatggcctgtaggctggagctgcttc<br>**R:**cagccatacctgaaccgggtactacatgtgtgatgattttcatatgaatatcctcctta	
MR32	Δ*prgH-orgC* Δ*invH-sipC*		P22

a. *All strains are based on Salmonella enterica serovar Typhimurium LT2 DW01.*
b. *Underlined sequences are priming site to template plasmid pKD3 (a) or pKD4 (b)* [6].
c. *Unless otherwise noted, the strain was constructed as part of this study. P22 indicates that the strain was built fusing P22 transduction.*

Supplementary Table 4: Point mutations to *spaO*

Location (nt)[a]	Mutation
496	tt**g** to tt**a**
499	ct**g** to ct**t**
529	ct**g** to ct**t**
532	at**t** to at**c**
606	at**t** to at**c**
609	gt**g** to gt**t**

a. Indicates the location from the first nucleotide of spaO.

Supplementary Table 5: RBS sequences designed for *spaOs*

Strength[a]	Sequence
1000	cttgggcacgcgtccataaggtatacgacccactag
2000	ttaggtcatttcaaccgtgttgaaggggaattatt
4500	cttgggcacgcgtccattaaatcgaggacccactag
5000 (a)	cttgggcacgcgtccatacctcgtaagacccactag
5000 (b)	gttatagtattttatagacgaggtattttatat
7500	cttgggcacgcgtccatatacggtaggacccactag
10000	taggtaggcgcccaccgaagaaagaggacacacgt
20000	accaaaacaagagaagagtaaggcataacaat

a. The designed translational initiation rate of the RBS in artificial units as predicted by the RBS Calculator. (a) and (b) refer to two designs of the same strength.

Supplementary Table 6: Genetic parts used in this study

Part Number	Part Type	Sequence
SBa_001697	T3.1 promoter	taataaccctcactacaggcaga
SBa_001698	K1F promoter	taataactatcactatagggaga
SBa_001749	J23100 promoter	ttgacggctagctcagtcctaggtacagtgctagct
SBa_001750	J23101 promoter	tttacagctagctcagtcctaggtattatgctagc
SBa_001737	J23110 promoter	tttacggctagctcagtcctaggtacaatgctagc
SBa_001738	P_{tac} promoter	gagctgttgacaattaatcatccggctcgtataatgtgtggaattgtgagcggataacaattctgctgctatag
SBa_001739	P_{BAD} promoter	agaaaccaattgtccatattgcatcagacattgccgtcactgcgtcttttactggctcttctcgctaaccaaaccggtaaccccgcttattaaa<br>agcattctgtaacaaagcgggaccaaagccatgacaaaaacgcgtaacaaaagtgtctataatcacggcagaaaagtccacattgattatttgc<br>acggcgtcacactttgctatgccatagcattttatccataagattagcggatcctacctg
SBa_001740	P_{tac-O2} promoter	gagctgttgacaattaatcatcggctcgtataatgtgtggaattgtgagcggataacaattcgacgtacggtggaaaattgtgagcggataaca<br>att
SBa_001741	P_{lux} promoter	acctgtaggatcgtacaggtttacgcaagaaaatggtttgttatagtcgaataaa
SBa_001752	RiboJ	agctgtcaccggatgtgctttccggtctgatgagtccgtgaggacgaaacagcctctacaaataattttgtttaa
SBa_001699	RBS	tactagagattaaagaggagaaattaagc
SBa_001700	RBS[a]	cctaggattaaggaggataaattaagc
SBa_001701	RBS[a]	gctagcattaaacaggataataagc
SBa_001702	RBS[a]	aagcttattaaacaggataataagc
SBa_001703	RBS[a]	ggtaccgaaagaagggacagactag
SBa_001704	RBS[a]	agatctgaaagaagggacagactag
SBa_001705	RBS	tactagagaaaaaaaagctctaaaagattaagagggggtaacat
SBa_001706	RBS	cttgggcacgcgtccattaagaaggaggaattaagc
SBa_001707	RBS	cgcttattcattcccgagacatcggagttagcc
SBa_001708	RBS[a]	gacgtccttgggcacgcgtccattaaacaggagtaattaagc
SBa_001709	RBS[a]	atttaaatgctagccttgggcacgcgtccattaaagaggaccaattaagc
SBa_001710	RBS	accaaaacaagagaagagtaaggcataacaat
SBa_001711	RBS	cttgggcacgcgtccatgaaagaaacgacatactag
SBa_001712	RBS[a]	gagctctgggcacgcgtccattaatgaggaaaaattattagc
SBa_001713	RBS	cttgggcacgcgtccatgaaagacaggacccactag
SBa_001714	RBS[a]	ctgcaggtttaaaccttgggcacgcgtccatgaaagacaggacccactag
SBa_001715	RBS[a]	agatctcttgggcacgcgtccattaaaaaggaccaattaagc
SBa_001742	RBS[a]	ggatccatcagataaacgcagtcgttaagttctacaaagtcggtgacagataacaggagtaagta
SBa_001743	RBS	agaaaaatttcgaaataaacagctcctactaaaaa
SBa_001744	RBS	tcacacaggaaaccggttcg
SBa_001751	RBS	tcacacaggaaag
SBa_001716	Codon optimized *prgI*	atggctactccgtggtctggttatctggatgatgtttctgcaaaatttgacacgggtgttgacaacttgcaaacccaagttaccgaagccctgg<br>acaagctggctgcgaagccgtccgatccggcgctgctggcggcgtatcaatccaaactgtctgagtataacttgtaccgtaatgcgcagtcgaa<br>cacggtcaaggtcttcaaagatattgatgcagcgattattcagaactttcgttagtaa
SBa_001717	Codon optimized *prgJ*	atgtctatcgcgactatcgtgcctgaaaatgccgttattggtcaagcggtgaatattcgcagcatggaaacggacatcgtcagcttggacgacc<br>gtttgctgcaagcattttcgggcagcgccatcgctaccgccgtcgataagcagaccattaccaatcgcattgaagaccctaatctggttaccga<br>tccgaaggagctggcgattagccaggaaatgatttccgactacaatctgtacgtcagcatggttagcaccctgacgcgtaagggcgttggcgct<br>gttgagactttgctgcgttcctgatag
SBa_001718	Codon optimized *prgH*	atggaaactagcaaggagaaaacgattacgtcccctggtccgtatatcgttcgtctgttgaactcgtccctgaacggttgtgaatttccgctgc<br>tgactggtcgtacgctgttcgtcgtgggtcagagcgatgctctgaccgcgtctggtcagctgccggacattcctgccgactccttcttcatccc<br>gctggatcatggcggtgttaatttcgagattcaagtggacactgacgcgacggaaatcatcctgcacgaactgaaagagggcaactccgagagc<br>cgctccgtgcaactgaacaccccgatccaagttggtgaactgctgattttgattcgtccggagagcgagccgtgggtgccggaacagccggaga<br>agttggaaacttctgcgaaaaagaacgaaccgcgctttaaaaacggcatcgtcgcggcactggcgggtttctttatcctgggtatcggcacggt<br>tggcaccctgtggattctgaactcgccgcaacgtcaagcagccgaattggacagcctgttgggtcaggagaaggagcgttttcaggtgctgccg<br>ggtcgcgacaagatgctgtatgtcgccgcgcaaaacgaacgcgacaccctgtgggcacgtcaagtcctggcacgcggcgattacgataaaaacg<br>cacgcgttattaatgaaaatgaggagaataaacgtatcagcatctggctggacacgtattatccacaactggcatattaccgtatccattttga<br>tgaaccacgtaagccggtgttttggctgtcccgtcaacgcaacacgatgagcaagaaagagctggaggtgctgtcccagaaattgcgtgcgctg<br>atgccgtacgccgacagcgtcaatattactctgatggatgacgtgaccgcagcaggccaagccgaggcaggtctgaaacaacaggcgctgccat<br>acagccgccgtaaccacaaaggtggtgttacgttcgttattcagggcgccttggacgacgttgagattctgcgtgcgcgccagtttgtcgactc<br>ctattatcgtacctggggtggtcgttacgttcaattcgcaattgaattgaaagacgattggctgaaaggccgctcgttccaatacggtgcggaa<br>ggctacattaagatgagcccaggtcattggtattttccgtctcctctgtaatag
SBa_001719	Codon optimized *prgK*	atgatccgccgttacctgtataccttcttgctggttatgactttggccggctgtaaagataaggatctgctgaaaggcttggaccaagagcaag<br>cgaatgaggtcattgcggttctgcaaatgcacaacattgaggctaacaagattgatagcggcaagctgggttacagcattaccgtcgcggaacc<br>ggatttcaccgcggcagtgtattggattaagacctaccagttgccgcctcgcccgcgtgtcgaaatcgcccaaatgtttccggcagacagcctg<br>gttagctctccgcgtgcggagaaagcacgtctgtactcggcgattgaacagcgcctggagcagtcgctgcaaacgatggaaggtgttctgtcgg<br>cccgtgtccacatcagctatgatattgatgcgggcgagaacggtcgtccgcctaagccggtgcacctgtcggctttggcggtgtatgaacgcgg<br>ttcccctctggcccatcagatttcggatattaagcgcttcctgaaaaacagcttcgcggatgttgactatgataacatcagcgtggttctgtcc<br>gagcgtagcgacgcacagttgcaggcgccgggcacgccggtcaagcgcaatagcttcgctacctcctggattgtgctgattatcctgttgtctg<br>ttatgagcgcgggtttcggtgtctggtactacaaaaatcactatgcgcgtaataagaaaggcattactgccgatgacaaggcaaagtccagcaa<br>cgagtaataa
SBa_001720	Codon optimized *orgA*	atgattcgtcgtaaccgtcaaatgaaccgtcaaccactgccaattatctggcaacgcattattttcgacccactgtcctatattcacccacaac<br>gtctgcaaatcgcgccggagatgatcgtgcgtccggcagcgcgtgcggcagcgaatgagctgattttggcggcgtggcgtttgaagaacggtga<br>gaaggagtgcattcagaatagcctgacgcagctgtggttgcgtcaatggcgccgtctgccgcaggttgcttacctgctgggttgccacaagttg<br>cgtgctgacctggcccgtcaaggtgctttattgggcctgccggactgggcgcaggcattcttggcgatgcaccagggtacgtccttgtcggttt<br>gtaataaggcgccgaaccaccgtttcctgctgtccgttggttacgcccaactgaacgcgctgaatgagttcctgccggagagcttggcccaacg<br>ctttcctctgctgtttccaccgttcatcgaggaggcactgaaacaagatgcagtggagatgagcatcctgctgctggccctgcaatacgcgcaa<br>aagtatcctaacaccgtcccggcgtttgcgtgctaataa

SBa_001721	Codon optimized *orgB*	atgttgaaaaatatcccgattccatcccctctgtctccggttgaaggtatcctgattaaacgcaagacgttggagcgttacttctcgattgagc<br>gcctggaacaacaggcgcatcagcgtgcaaagcgcattttgcgtgaggcagaagaagaagccaagaccctgcgcatgtatgcgtaccaggaggg<br>ctacgagcagggcatgattgacgcactgcaacaggtggccgcctatttgaccgacaaccagacgatggcttggaaatggatggagaaaattcaa<br>atctatgcgcgtgagttgttcagcgcggctgtcgatcacccggaaacgttgttgacggtgctggacgagtggctgcgtgatttcgataagccgg<br>aaggtcagctgtttttgaccctgccggtgaacgcaaagaaagatcatcagaaactgatggtgctgctgatggaaaattggccgggcaccttcaa<br>tctgaagtatcatcaggagcaacgttttatcatgtcctgtggcgatcagattgccgagttttcgccggaacaatttgttgaaacggcggttggt<br>gttatcaagcaccatctggatgagctgcctcaggactgtcgcaccatttcggacaatgcgattaacgcgctgattgatgaatggaagacgaaaa<br>cccaagctgaagttattcgctgataa
SBa_001722	Codon optimized *invJ*	atgggtgacgtgagcgcggtgagcagcagcggtaacattctgctgccgcagcaggacgaggttggtggcctgtccgaagcgctgaagaaagcgg<br>ttgaaaaacacaaaaccgaatacagcggtgacaagaaagatcgtgattatggtgacgcctttgttatgcacaaggaaaccgcgctgccgttgtt<br>gctggcagcttggcgccacggcgcaccggcgaaaagcgagcaccataacggtaacgtaagcggtctgcatcacaacggtaagagcgagctgcgt<br>attgctgagaaactgctgaaggtgacggcggagaagagcgttggtctgattagcgctgaagcgaaggtggataaatctgcggcgctgctgtcta<br>gcaagaatcgtccgctggaatcggtcagcggcaaaaagttgtccgccgatctgaaagcagtggagtccgtgtccgaggtcacggataacgccac<br>cggcatttcggatgacaacatcaaagcattgccgggtgacaataaggccatcgccggtgagggtgtgcgtaaagaaggtgcgccgctggcgcgt<br>gacgtggctccggcacgcatggcggcagcaaatacgggcaagccggaggataaagaccacaagaaggtcaaggacgttagccagctgccgctgc<br>aaccgactaccatcgccgatctgtctcaactgacgggtggcgatgaaaagatgccgctggcagcgcagtccaaaccgatgatgaccatttccc<br>aaccgccgacggcgttaaaggtgaggacagctctctgacctatcgtttccagcgctggggcaacgattactccgtcaatatccaggcacgccaa<br>gcgggcgaatttagcctgattcctagcaatacccaggttgaacatcgtctgcacgaccaatggcaaaatggcaatccacaacgctggcatttga<br>cgcgtgatgaccagcaaaacccgcaacagcaacagcatcgtcagcagtccggtgaagaggacgacgcgtaa
SBa_001723	Codon optimized *spaQ*	atggacgatctggttttcgccggcaacaaagccctgtacttggtgctgattctgtccggttggccgacgattgtcgcaaccattatcggtctgc<br>tggttggtctgtttcaaaccgtgacgcagttgcaggagcaaaccctgccgttcggtatcaagctgctgggtgtgtgtctgtgcctgtttttgct<br>gtctggctggtatggcgaagttctgctgtcctacggccgtcaggtcatcttcctggctctggcgaaaggttaa
SBa_001724	Codon optimized *invH*	atgaaaaagttctacagttgtctgcccgtctttcttttgataggttgcgcacaagtaccgctaccgtcgtctgtgtccaagccggttcagcaac<br>cgggagcgcagaaagaacaactggctaacgccaatagcattgatgagtgccagagcctgccttatgtcccaagcgacctggccaaaaacaaatc<br>cttatcaaaccataatgccgacaattcagcgtctaaaaactcggcgattagcagctcgatcttttgtgagaaatacaagcaaacgaaagaacag<br>gcgctgaccttctttcaggaacacccgcagtatatgcgtagtaaagaggatgaagaacagttaatgactgagttcaaaaaggtgctcctggaac<br>ctggctccaaaaatctgagtatctatcagaccctgcttgccgcacatgaacgcttgcaggcgctctaa
SBa_001725	Codon optimized *invA*	atgctgctgtccctgctgaatagcgcgcgtctgcgtcctgagctgctgattctggttctgatggttatgatcatcagcatgttcgttatcccgt<br>tgccgacctatttggttgacttcttgatcgctttgaacattgtcctggcaattctggtgttcatgggctccttctacatcgaccgcattctgag<br>cttcagcacctttccggcggttctgctgatcacgactctgttccgtttggcactgagcatcagcaccagccgcctgatcctgattgaagcagat<br>gcgggtgagatcatcgcgacctttggtcagtttgtgatcggtgacagcctggcggttggtttcgtcgtattctccatcgtgacggtggtgcagt<br>ttatcgttattaccaagggcagcgaacgtgtggcggaggtcgccgctcgcttcagcctggacggcatgccgggtaaacagatgtctattgatgc<br>agacctgaaagccggcattattgatgctgatgcagcgcgcgagcgccgcagcgtcctggagcgtgaaagccaactgtacggttccttcgacggt<br>gccatgaagttcattaaaggtgatgcgattgcgggcatcattatcatcttcgttaacttcattggcggtatcagcgtcggtatgacccgtcatg<br>gtatggatctgagcagcgccctgagcacctacaccatgctgacgattggtgatggtctggttgcccaaattccggcgttgctgatcgcgatttc<br>tgcgggcttcatcgttacccgcgtcaacggtgatagcgataacatgggtcgtaacattatgacccagctgctgaataatccgtttgtcctggtt<br>gtaacggcgattttgaccatcagcatgggcacgctgccgggctttccgttgccggttttcgttattctgtctgttgtgctgtccgtcctgtttt<br>actttaagttccgcgaggcgaaacgtagcgctgcgaaaccaaaaacgagcaagggcgagcaaccgttgtccatcgaggagaaggaaggtagcag<br>cctgggcctgattggcgacctggataaggttagcacggaaaccgtcccgctgattttgctggtgccgaaatcgcgtcgtgaggatctggagaaa<br>gcgcagctggcggaacgtctgcgcagccaattctttattgattatggtgtgcgtctgccagaagtactgctgcgtgacggtgagggtctggatg<br>acaactctatcgtcctgctgattaatgagattcgcgttgaacagtttactgtctattttgacctgatgcgtgtggttaactacagcgacgaggt<br>ggtgagctttggcatcaacccgaccattcaccagcaaggttccagccagtacttttgggtgacccatgaggaaggcgaaaagctgcgcgagctg<br>ggctacgtcctgcgtaatgcactggacgaactgtaccactgtctggcggtgacgctggcacgcaatgtgaacgagtatttcggtatccaagaaa<br>cgaaacacatgctggaccaactggaagcaaagtttcctgacctgctgaaggaggttttgcgccacgccaccgtgcagcgcatttcggaagtgct<br>gcaacgtctgctgtccgaacgcgtgagcgtccgtaacatgaagctgatcatggaagccctggcactgtgggctccgcgtgagaaagatgtgatc<br>aatctggtggagcacatccgtggtgcgatggcgcgttatatctgccacaagttcgcaaatggtggtgaactgcgtgccgttatggtttccgccg<br>aagttgaggatgtcattcgtaaaggcattcgtcaaacttctggctccacctttttgagcttggacccggaggcttcggcaaatctgatggacct<br>gatcacgctgaagctggacgacctgttgattgcgcataaggacctggtcctgttgaccagcgttgacgtgcgtcgttttatcaagaaaatgatt<br>gaaggtcgttttccggatctggaggtcctgtccttcggtgagattgcagatagcaaaagcgtgaatgtcatcaaaccatctga
SBa_001726	Codon optimized *invC*	atgaaaaccccacgtctgctgcaatacctggcctacccgcagaaaatcactggccctatcattgaagcagaactgcgtgatgttgcaattggtg<br>aattgtgcgagatccgtcgcggctggcaccagaagcaggttgtggcccgtgcgcaagtggttggtttgcagcgcgaacgtaccgtcctgagcct<br>gatcggcaatgcccaaggcctgagccgtgatgtggtcttgtacccgaccggccgtgctctgagcgcgtgggttggttacagcgttctgggcgca<br>gtactggacccgacgggtaaaatcgttgaacgtttcaccccggaagtcgcaccgatttccgaggagcgcgttatcgacgtggcaccgccgagct<br>acgcaagccgtgtcggtgtgcgcgaaccgctgatcacgggtgtccgcgcaattgatggtctgctgacgtgtggtgtgggccagcgtatgggtat<br>tttcgcaagcgcgggttgtggtaagaccatgttgatgcacatgctgatcgagcaaaccgaagcggatgtctttgttattggcctgattggcgag<br>cgtggtcgtgaagttaccgaatttgtagacatgttgcgtgcatctcataagaaagaaaaatgtgtgctggtttttgccacgtccgacttcccaa<br>gcgttgaccgctgcaacgctgcccagctggcaacgacggtcgccgagtatttccgcgaccagggtaaacgtgttgtcctgtttatcgacagcat<br>gacccgttatgcacgcgcgttgcgtgatgtcgcgctggcgagcggcgagcgtccggctcgccgtggttatccggcgtctgtgttcgacaatctg<br>ccgcgtttgctggagcgtccgggtgcgacgagcgagggtagcattaccgccttctataccgtcctgctggagtcggaagaagaagcggacccga<br>tggcggacgagatccgttctattctggacggtcacctgtacctgtcccgcaaactggcgggtcagggtcattacccggctatcgatgtgctgaa<br>gagcgtgagccgtgtgtttggtcaagtgaccaccccgactcacgcggagcaagcgagcgcggtccgtaagctgatgacccgtctggaagagctg<br>caactgttcattgacctgggcgagtatcgtccgggcgagaacattgacaatgatcgtgcgatgcaaatgcgcgatagcctgaaggcgtggctgt<br>gtcagcctgttgcgcaatacagcagcttcgatgatacgctgtccggcatgaacgcctttgcggatcagaactga
SBa_001727	Codon optimized *spaOs*	atggaaaccctggacatccaacacattgaagaagagaacaacacgaccgaaaccgcggaaaccctgccgggtctgaaccagctgccggttaaac<br>tggagtttgtactgtaccgtaagaatgtgaccctggcggagctggaggcgatgggtcagcagcaactgctgtctctgccgacgaacgcagagtt<br>gaatgtggaaatcatggccaacggcgtcctgctgggtaatggtgagttggtccagatgaacgataccctgggtgttgaaattcacgagtggctg<br>tctgagagcggcaatggtgagtga
SBa_001728	Codon optimized *spaP*	atgggtaacgatattagcttgattgcattgctggcgttttccaccctgctgccgttcatcatcgcgtctggtacttgcttcgtcaaattcagca<br>tcgtctttgtgatggtgcgcaacgcgctgggtctgcaacaaattccaagcaatatgaccctgaatggcgtcgcactgctgctgtcgatgtttgt<br>tatgtggccgattatgcacgacgcgtatgtgtatttcgaggatgaagatgtgacctttaacgacatctccagcctgagcaagcatgttgatgag<br>ggcctggacggttatcgcgactacctgatcaagtattccgaccgtgagctggtgcagttctttgagaatgcccagttgaaacgtcagtacggtg<br>aagaaacggaaaccgttaaacgtgacaaggacgagattgaaaagccgagcattttcgcactgttgcctgcttacgccttgagcgagattaagag<br>cgcattcaaaattggtttttacctgtacctgccgttcgttgtggtcgatctggttgtctccagcgttctgctggccctgggcatgatgatgatg<br>tccccggttaccatcagcacgccgatcaaactggtcctgtttgtggccctggatggttggacgctgctgtctaaaggcctgatcctgcaataca<br>tggacatcgcgacctaa
SBa_001731	Codon optimized *invG*	atgaaaacgcacattctgttggcccgtgtgctggcttgcgcagctctggtgctggtcaccccaggttatagctccgagaagatcccggttacgg<br>gcagcggcttcgttgcaaaggacgattctctgcgcaccttttcgatgcgatggcactgcaattgaaggagccggtgattgtcagcaagatggc<br>ggctcgcaaaaagattaccggcaatttcgagttccacgatccaaacgcgctgctggagaaactgtccctgcaactgggtctgatctggtacttt<br>gatggtcaggcgatctacatctacgacgcgagcgaaatgcgtaatgcggttgtgagcctgcgtaatgtcagcctgaacgagttcaacaattttc<br>tgaagcgcagcggcctgtacaataagaactaccctctgcgtggtgataatcgtaaaggcaccttctatgtcagcggtccgccggtgtatgttga<br>tatggttgtaaatgcggccaccatgatggacaaacagaatgatggcatcgagctgggtcgccaaaagatcggtgttatgcgcctgaacaacact<br>tttgtgggcgaccgcacctacaacctgcgtgatcaaaagatggtcattccgggtattgctacggcaattgaacgcctgttgcaaggcgaagaac<br>aaccgctgggtaacattgtaagctccgagcctccggccatgccggcctttagcgcaaacggcgagaaaggtaaggcagcgaattacgcgggtgg<br>tatgagcctgcaagaagcgctgaaacagaacgcagcggcaggcaacatcaaaattgtggcctatccggacaccaacagcctgctggtgaaaggt<br>acggcggagcaggtgcatttcatcgagatgctggttaaagccctggacgtggcgaaacgtcacgttgaattgagcctgtggattgtggatttga<br>ataagagcgacctggaacgcttgggcaccagctggagcggtagcatcaccatcggcgacaagctgggtgtgagcctgaaccagagcagcatctc<br>cacgctggacggtagccgctttattgcggcggtcaacgctctggaggaaaagaaacaggccactgttgtcagccgtccggttctgctgacccag<br>gagaatgtcccggcgattttgacaataatcgtactttctataccaaactgatcggtgaacgtaatgttgcattggaacacgtgacctatggca<br>ccatgatccgtgtcctgccgcgtttcagcgcggacggccagattgagatgagcctggacatcgaagatggtaatgacaaaaccccgcagtctga<br>tacgaccacctccgttgatgcgctgccagaagtgggtcgtaccctgatctcgacgattgcacgtgtcccgcatggtaaatctctgctggttggt<br>ggctacacgcgtgatgcaaacacggacacggtccaaagcatcccgttcctgggtaagctgccgctgattggctcgttgtttcgctacagcagca<br>aaaacaagtctaatgtcgtccgtgtctttatgattgagccgaaagaaatcgttgacccgctgaccccggatgccagcgagagcgttaacaacat<br>tctgaaacagtccggtgcgtggagcggtgacgataagctgcaaaagtgggttcgtgtgtatttggaccgtggtcaggaggccattaagtaa

SBa_001732	Codon optimized *spaS*	atgtccagcaacaaaaccgaaaaaccgactaagaaacgtctggaggatagcgcaaagaaaggtcagagcttcaagagcaaggacctgattatcg<br>cgtgcctgaccctgggtggtatcgcttatttggtgagctacggcagcttcaatgagtttatgggtatcattaagattatcatcgctgataactt<br>tgatcagtcgatggcagattatagcctggccgtgtttggtattggcctgaaatacctgattccgttcatgctgctgtgtttggtttgttccgca<br>ctgccggcactgctgcaagcgggcttcgttctggcaaccgaggccctgaagccgaatctgtccgccctgaacccggttgaaggcgcgaagaaac<br>tgttttccatgcgcaccgtcaaagacacggtcaagacgctgctgtatctgtcgagctttgtggttgcggcaatcatttgctggaaaaagtataa<br>agtcgagattttcagccaactgaacggtaatatcgtgggtattgcggttatctggcgtgaattgctgctggcgttggttctgacctgtctggcg<br>tgcgcgctgatcgtgctgttgctggatgctattgccgagtactttctgaccatgaaagatatgaagatggacaaagaagaagttaaacgcgaga<br>tgaaagagcaggagggtaacccggaggtgaagtctaaacgtcgtgaagtccacatggaaatcctgagcgaacaagtcaagtctgacattgaaaa<br>tagccgtctgattgtggcaaaccctacgcatattaccatcggcatctacttcaaaccggaactgatgccgattccaatgattagcgtctatgaa<br>accaatcaacgcgcgctggcggtccgtgcgtacgccgagaaagtgggtgttccggttattgtagacatcaagctggcgcgcagcctgttcaaaa<br>cgcaccgtcgttacgacctggtgagcctggaggagatcgacgaggttttgcgcctgctggtttggttggaagaggtcgaaaacgcaggcaagga<br>tgtgatccaaccgcaggagaatgaagtgcgtcattaa
SBa_001733	Codon optimized *spaO*	atgagcttgcgcgtacgccaaattgaccgccgtgaatggctgctggcccagactgcgaccgaatgtcagcgtcacggtcgtgaggccaccctgg<br>agtatccgacccgtcagggtatgtgggtccgcctgtctgatgccgaaaaacgctggtctgcgtggatcaaaccgggtgattggttggaacacgt<br>tagcccagcactggcgggtgccgcggtcagcgcaggcgcggagcacctggtggttccgtggctggcggcaaccgaacgcccgttcgagctgccg<br>gtcccgcacttgagctgccgtcgtctgtgcgtggagaacccggttccgggttccgcactgcctgagggcaagctgctgcacatcatgtcggatc<br>gtggtggcctgtggtttgagcatctgccggagctgccggctgttggcggtggccgtccgaagatgctgcgttggccgctgcgtttcgttattgg<br>cagcagcgacacccagcgcagcctgctgggtcgtatcggtatcggtgatgttctgctgatccgcaccagccgtgctgaggtttactgttacgcg<br>aagaaactgggccactttaatcgtgtggaaggtggcattattgtcgaaacgctggacattcaacatatcgaggaggagaacaacacgacggaaa<br>cggcggaaaccctgccgggtctgaatcaactgccggtgaagctggagttcgttctgtatcgtaaaaacgtgacgttggccgaactggaagcaat<br>gggtcagcaacaactgctgtccttgccaaccaatgcggaactgaacgtcgaaatcatggcgaatggcgtgctgctgggtaacggcgaactggtg<br>cagatgaatgacaccctgggtgtcgagattcatgagtggttgtccgagagcggtaatggcgagtag
SBa_001734	Codon optimized *invI*	atgcactctctgactcgtatcaaggtcctgcaacgtcgttgtaccgtgttccattctcagtgcgagtccattctgttgcgttatcaggacgaag<br>atcgcggcttgcaggcggaggaggaggccatcctggaacagatcgcaggtctgaaactgctgttggacaccctgcgtgctgaaaatcgtcaact<br>gagccgtgaagaaatctatacgctgctgcgcaaacagagcattgttcgtcgccagattaaggatctggagctgcaaatcattcagattcaagaa<br>aagcgtagcgagctggaaaagaaacgtgaggaatttcaaaagaaaagcaaatactggctgcgtaaagaaggtaactaccagcgctggattatcc<br>gtcaaaaacgcttctacattcaacgtgagatccagcaagaggaggcggagagcgaagagatcatttaa
SBa_001735	Codon optimized *spaR*	atgttctatgcattgtattttgagatccaccatctggtggcgtccgcggctctgggttttgcgcgtgttgctccgatctttttctttctgccgt<br>tcctgaatagcggtgtcctgagcggtgctccgcgcaacgcgatcatcattctggtggcgctgggtgtatggccgcacgccctgaatgaggcgcc<br>accgtttctgtctgtggcaatgattccgctggtcctgcaagaggcagccgtgggtgttatgctgggttgcctgttgtcctggccgttttgggtt<br>atgcacgcgttgggctgtatcattgataaccaacgcggtgcaaccctgtccagcagcattgatcctgcgaatggcatcgacaccagcgagatgg<br>ccaatttcctgaacatgttcgcggctgtcgtgtatttgcagaacggtggcttggtcacgatggtggacgtgctgaataagtcttaccagctgtg<br>tgatccgatgaacgagtgtacgccgagcctgcctccgttgctgaccttcattaatcaagtggcccagaacgcactggtgctggcgtccccggtc<br>gttctggttctgctgctgtcggaagttttcctgggcctgctgtctcgttttgcaccgcaaatgaacgcgttcgccattagcctgactgttaaaa<br>gcggtattgcggttttgatcatgctgctgtatttcagcccggtcctgccggacaatgttctgcgtctgagctttcaggcgaccggcctgagcag<br>ctggttctacgaacgtggcgcaacgcatgtgctggaataa
SBa_001753	Codon optimized *invF*	atgagcttcagcgagagccgccacaatgaaaactgtctgattcaagaaggcgcactgctgttttgtgagcaagcagtcgtggcgcctgtcagcg<br>gtgatctggtttttcgtccgctgaaaatcgaggtcctgagcaagctgctggcgttcatcgacggcgcaggtctggtggatacgacctacgcgga<br>gtcggacaaatgggttctgctgtctccggagttccgtgctatttggcaagaccgtaaacgttgcgaatattggtttttgcagcagattatcacc<br>ccatctccggcgttcaacaaggttctggcactgttgcgtaagagcgaaagctattggttggtcggctacttgctggcccaaagcaccagcggca<br>atactatgcgtatgttgggtgaggattacggtgttagctacacgcatttccgccgtctgtgcagccgcgctctgggcggtaaggcgaaaagcga<br>gctgcgcaattggcgcatggcccagtccctgctgaatagcgtggaaggtcatgaaaacatcacccagctggcggtcaaccacggttatagcagc<br>ccgtcccactttagctctgaaatcaaggagctgattggtgtttccccgcgtaagctgtctaacatcattcagctggccgacaaatga
SBa_001754	Codon optimized *invE*	atgattccgggcagcacctccggtatttcctttagccgtatcctgagccgtcagacctcccaccaggatgcaacccagcataccgacgcacaac<br>aagcggaaattcaacaagcggcggaagatagctcgccgggtgcggaggttcagaaattcgtccagagcacggacgagatgtctgctgcgttggc<br>gcagttccgcaatcgccgtgactatgagaaaaagagcagcaatttgtctaactccttcgagcgcgttctggaggacgaggcactgccgaaagcg<br>aagcagattctgaaactgatcagcgtgcatggcggtgcgttggaggatttcctgcgtcaggcgcgcagcctgttcccggacccaagcgatctgg<br>tgctggttctgcgcgagctgttgcgtcgtaaggacctggaggagatcgtgcgtaagaagctggagagcctgctgaagcacgtggaggaacaaac<br>cgacccgaaaaccctgaaggccggtattaactgcgcgctgaaggcgcgtctgtttggcaagacgctgtctctgaaacctggtctgctgcgtgcc<br>agctaccgccagttcatccaaagcgaaagccacgaagtcgagatttacagcgattggatcgccagctacggttatcagcgtcgcctggttgttc<br>tggatttcattgaaggcagcctgctgactgacatcgatgctaacgatgcaagctgctcccgtctggagtttggccaactgctgcgccgtctgac<br>ccagctgaaaatgttgcgtagcgccgacctgctgtttgtctcgacgttgctgtcttacagcttcacgaaagcatttaacgctgaggagagcagc<br>tggctgttgctgatgctgtctttgctgcaacagccgcacgaagtggatagcctgctggcggacattatcggtctgaatgcgctgctgttgtccc<br>acaaagagcacgccagcttcctgcaaatcttctatcaggtctgtaaggcaatcccgtctagcctgttttatgaagagtactggcaagaagaact<br>gctgatggcactgcgctccatgacggacattgcttacaaacacgaaatggccgaacaacgtcgtaccatcgaaaagctgtcctaa
SBa_001755	invR	agctatggtcacttttacggttggccatttgtctcttacgttgcatttatcaatctgcttttgatacaGCAGCACCTCGCTGCTGCttttttt
SBa_001756	Terminator	ggcatcaaataaaacgaaaggctcagtcgaaagactgggcctttcgttttatctgttgtttgtcggtgaacgctctcctgagtaggacaaatcc<br>gccgccctaga
SBa_001757	Terminator	ccaggcatcaaataaaacgaaaggctcagtcgaaagactgggcctttcgttttatctgttgtttgtcggtgaacgctctc
SBa_001758	Terminator	ccggcttatcggtcagtttcacttcttcataaaacccgcttcggcgggtttttgcttttacagggcggcaggatgaatgactgtccacgacgct<br>atacccaaaagaaa
SBa_001745	SicP-SptP-FLAG	atgcaagcacaccaggatattatcgctaatattggtgagaaattgggtttaccgctcacttttgacgacaacaatcagtgcttattattactcg<br>atagcgatattttacgtctattgaagctaaagatgatatctggttattgaacggtatgattataccgttatcgcctgtttgtggcgattctat<br>ctggcggcagattatggtgattaatggtgaactggctgcgaataatgaaggtacgttagcgtatattgatgccgcagagacgttgttgcttata<br>catgcaattaccgatctgacaaatacttaccatattatatcgcagcttgagtcatttgtgaatcagcaggaagcgctcaaaaacatactgcagg<br>aatatgctaaagtatgaggagagaaaattgaataatttaacgttgtcttcgttttcaaaagttggtgtgtcgaatgatgcccgactttatattg<br>ctaaggaaaatactgataaggcatatgttgcgcctgaaaaattttcgtcaaaagtattaacctggcttggaaaaatgccgttatttaaaaacac<br>tgaagtggtgcaaaaacatacggaaaatatcagagtacaggaccaaaagatttacagacatttctccatgcactaacggaaaaatatggggaa<br>acagcggttaatgacgcactgttaatgtcccgtataaatatgaacaaaccccttacccaacgtttagcagtgcagatcacggagtgtgtaaaag<br>ctgctgacgaagggtttataaaccttattaagagcaaggataatgttggtgtcaggaatgccgctttagtcataaaggcggcgatacaaaagt<br>ggcagaaaaaaataacgatgttggagcagaaagtaagcaacctttactcgatatcgcgctaaagggacttaaaagaacattaccgcaactggaa<br>caaatggatggaaatagcctgcgagaaaacttccaggagatggcttcaggtaacggcccgctgcgttcattaatgacgaatttacagaatttaa<br>ataaaattccagaggctaaacaacttaatgattatgttacaacgttaacaaatattcaggtcggtgtagcccggttctcacaatggggaacctg<br>tggaggagaggttgagaggtgggttgataaagcctctactcatgaattaacccaggctgtaaaaaaattcacgttattgctaaagaactgaaa<br>aatgttacagctgaacttgagaaaatagaagccggcgcgccaatgccacagacgatgagcggaccgacactgggcctggcacgatttgcggtca<br>gcagcattccaattaatcagcaaacccaggtgaaactgagcgatggaatgcctgtgccagtgaatacgttaacttttgacggtaagcctgtggc<br>attagccggttcgtacccaaaaaatacgccggatgcgctggaagcgcatatgaaaatgcttcttgaaaaagaatgctcgtgcctggtggtgtta<br>acgtcggaagatcagatgcaggcaaaacaattaccaccctatttcagagggagctacacctttggcgaggtgcataccaacagccaaaaagtga<br>gctcagccagtcagggagaagcgatagaccaatacaatatgcaactgtcctgcggggaaaagcggtatacaatcccggtattgcatgtgaaaaa<br>ttggccagatcaccagccgttaccgtctacggatcagttagaatacctggcggatagggtgaaaaatagtaaccaaaatggcgcgccggggcgt<br>agttcatcagataagcatttaccgatgattcattgtctgggcggagtgggaagaaccggaacgatggcggcggcccttgtacttaaggataatc<br>ctcatagtaatctggagcaggtacgtgcagatttccgggattcacggaacaatagaatgctggaagacgcctcacagtttgtacaactaaaagc<br>aatgcaagcccagttgcttatgacgacggcaagctctagagattataaagatgacgatgacaaggattataaagatgacgatgacaaggattat<br>aaagatgacgatgacaagtaa

SBa_001746	T7*(T3)	atgaacacgattaacatcgctaagaacgacttctctgacatcgaactggctgctatcccgttcaacactctggctgaccattacggtgagcgtt<br>tagctcgcgaacagttggcccttgagcatgagtcttacgagatgggtgaagcacgcttccgcaagatgtttgagcgtcaacttaaagctggtga<br>ggttgcggataacgctgccgccaagcctctcatcactaccctactccctaagatgattgcacgcatcaacgactggtttgaggaagtgaaagct<br>aagcgcggcaagcgcccgacagccttccagttcctgcaagaaatcaagccggaagccgtagcgtacatcaccattaagaccactctggcttgcc<br>taaccagtgctgacaatacaaccgttcaggctgtagcaagcgcaatcggtcgggccattgaggacgaggctcgcttcggtcgtatccgtgacct<br>tgaagctaagcacttcaagaaaaacgttgaggaacaactcaacaagcgcgtagggcacgtctacaagaaagcatttatgcaagttgtcgaggct<br>gacatgctctctaagggtctactcggtggcgaggcgtggtcttcgtggcataaggaagactctattcatgtaggagtacgctgcatcgagatgc<br>tcattgagtcaaccggaatggttagcttacaccgccaaaatgctggcgtagtaggtcaagactctgagactatcgaactcgcacctgaatacgc<br>tgaggctatcgcaacccgtgcaggtgcgctggctggcatctctccgatgttccaaccttgcgtagttcctcctaagccgtggactggcattact<br>ggtggtggctattgggctaacggtcgtcgtcctctggcgctggtgcgtactcacagtaagaaagcactgatgcgctacgaagacgtttacatgc<br>ctgaggtgtacaaagcgattaacattgcgcaaaacaccgcatggaaaatcaacaagaaagtcctagcggtcgccaacgtaatcaccaagtggaa<br>gcattgtccggtcgaggacatccctgcgattgagcgtgaagaactcccgatgaaaccggaagacatcgacatgaatcctgaggctctcaccgcg<br>tggaaacgtgctgccgctgctgtgtaccgcaaggacaaggctcgcaagtctcgccgtatcagccttgagttcatgcttgagcaagccaataagt<br>ttgctaaccataaggccatctggttcccttacaacatggactggcgcggtcgtgtttacgctgtgtcaatgttcaacccgcaaggtaacgatat<br>gaccaaaggactgcttacgctggcgaaaggtaaaccaatcggtaaggaaggttactactggctgaaaatccacggtgcaaactgtgcgggtgtc<br>gacaaggttccgttccctgagcgcatcaagttcattgaggaaaaccacgagaacatcatggcttgcgctaagtctccactggagaacacttggt<br>gggctgagcaagattctccgttctgcttccttgcgttctgctttgagtacgctggggtacagcaccacggcctgagctataactgctcccttcc<br>gctggcgtttgacgggtcttgctctggcatccagcacttctccgcgatgctccgagatgaggtaggtggtcgcgcggttaacttgcttcctagt<br>gaaaccgttcaggacatctacgggattgttgctaagaaagtcaacgagattctacaagcagacgcaatcaatgggaccgataacgaagtagtta<br>ccgtgaccgatgagaacactggtgaaatctctgagaaagtcaagctgggcactaaggcactggctggtcaatggctggcttacggtgttactcg<br>cagtgtgactaagcgttcagtcatgacgctggcttacgggtccaaagagttcggcttccgtcaacaagtgctggaagataccattcagccagct<br>attgattccggcaagggtctgatgttcactcagccgaatcaggctgctggatacatggctaagctgatttgggaatctgtgagcgtgacggtgg<br>tagctgcggttgaagcaatgaactggcttaagtctgctgctaagctgctggctgctgaggtcaaagataagaagactggagagattcttcgcaa<br>gcgttgcgctgtgcattgggtaactcctgatggtttccctgtgtggcaggaatacaagaagcctattcagaagcgcctggacatgattttcttg<br>ggtcaatttcgcttgcaacctaccattaacaccaacaaagatagcgagattgatgcacacaaacaggagtctggtatcgctcctaactttgtac<br>acagccaagacggtagccaccttcgtaagactgtagtgtgggcacacgagaagtacggaatcgaatcttttgcactgattcacgactccttcgg<br>tacgattccggctgacgctgcgaacctgttcaaagcagtgcgcgaaactatggttgacacatatgagtcttgtgatgtactggctgatttctac<br>gaccagttcgctgaccagttgcacgagtctcaattggacaaaatgccagcacttccggctaaaggtaacttgaacctccgtgacatcttagagt<br>cggacttcgcgttcgcgtaa
SBa_001747	T7*(K1F)	atgaacacgattaacatcgctaagaacgacttctctgacatcgaactggctgctatcccgttcaacactctggctgaccattacggtgagcgtt<br>tagctcgcgaacagttggcccttgagcatgagtcttacgagatgggtgaagcacgcttccgcaagatgtttgagcgtcaacttaaagctggtga<br>ggttgcggataacgctgccgccaagcctctcatcactaccctactccctaagatgattgcacgcatcaacgactggtttgaggaagtgaaagct<br>aagcgcggcaagcgcccgacagccttccagttcctgcaagaaatcaagccggaagccgtagcgtacatcaccattaagaccactctggcttgcc<br>taaccagtgctgacaatacaaccgttcaggctgtagcaagcgcaatcggtcgggccattgaggacgaggctcgcttcggtcgtatccgtgacct<br>tgaagctaagcacttcaagaaaaacgttgaggaacaactcaacaagcgcgtagggcacgtctacaagaaagcatttatgcaagttgtcgaggct<br>gacatgctctctaagggtctactcggtggcgaggcgtggtcttcgtggcataaggaagactctattcatgtaggagtacgctgcatcgagatgc<br>tcattgagtcaaccggaatggttagcttacaccgccaaaatgctggcgtagtaggtcaagactctgagactatcgaactcgcacctgaatacgc<br>tgaggctatcgcaacccgtgcaggtgcgctggctggcatctctccgatgttccaaccttgcgtagttcctcctaagccgtggactggcattact<br>ggtggtggctattgggctaacggtcgtcgtcctctggcgctggtgcgtactcacagtaagaaagcactgatgcgctacgaagacgtttacatgc<br>ctgaggtgtacaaagcgattaacattgcgcaaaacaccgcatggaaaatcaacaagaaagtcctagcggtcgccaacgtaatcaccaagtggaa<br>gcattgtccggtcgaggacatccctgcgattgagcgtgaagaactcccgatgaaaccggaagacatcgacatgaatcctgaggctctcaccgcg<br>tggaaacgtgctgccgctgctgtgtaccgcaaggacaaggctcgcaagtctcgccgtatcagccttgagttcatgcttgagcaagccaataagt<br>ttgctaaccataaggccatctggttcccttacaacatggactggcgcggtcgtgtttacgctgtgtcaatgttcaacccgcaaggtaacgatat<br>gaccaaaggactgcttacgctggcgaaaggtaaaccaatcggtaaggaaggttactactggctgaaaatccacggtgcaaactgtgcgggtgtc<br>gacaaggttccgttccctgagcgcatcaagttcattgaggaaaaccacgagaacatcatggcttgcgctaagtctccactggagaacacttggt<br>gggctgagcaagattctccgttctgcttccttgcgttctgctttgagtacgctggggtacagcaccacggcctgagctataactgctcccttcc<br>gctggcgtttgacgggtcttgctctggcatccagcacttctccgcgatgctccgagatgaggtaggtggtcgcgcggttaacttgcttcctagt<br>gaaaccgttcaggacatctacgggattgttgctaagaaagtcaacgagattctacaagcagacgcaatcaatgggaccgataacgaagtagtta<br>ccgtgaccgatgagaacactggtgaaatctctgagaaagtcaagctgggcactaaggcactggctggtcaatggctggcttacggtgttactcg<br>cagtgtgactaagcgttcagtcatgacgctggcttacgggtccaaagagttcggcttccgtcaacaagtgctggaagataccattcagccagct<br>attgattccggcaagggtctgatgttcactcagccgaatcaggctgctggatacatggctaagctgatttgggaatctgtgagcgtgacggtgg<br>tagctgcggttgaagcaatgaactggcttaagtctgctgctaagctgctggctgctgaggtcaaagataagaagactggagagattcttcgcaa<br>gcgttgcgctgtgcattgggtaactcctgatggtttccctgtgtggcaggaatacaagaagcctattcagacgcgcttgaacctgatgttcctc<br>ggttcgttcaacctccagccgaccgtcaacaccaacaaagatagcgagattgatgcacacaaacaggagtctggtatcgctcctaactttgtac<br>acagccaagacggtagccaccttcgtaagactgtagtgtgggcacacgagaagtacggaatcgaatcttttgcactgattcacgactccttcgg<br>tacgattccggctgacgctgcgaacctgttcaaagcagtgcgcgaaactatggttgacacatatgagtcttgtgatgtactggctgatttctac<br>gaccagttcgctgaccagttgcacgagtctcaattggacaaaatgccagcacttccggctaaaggtaacttgaacctccgtgacatcttagagt<br>cggacttcgcgttcgcgtaa

a. Underlined sequences are restriction sites that are a legacy of the initial design.

Supplementary Note 1: Supplementary data used to refactor SPI-1

1.A. Gene order and RBS selection

RBS sequences were selected for each gene to approximately match the ratios of protein that are expressed in the T3SS needle structure. This was gleaned using various sources of information and datasets containing protein numbers became available over the course of the project as well as new tools for RBS design and characterization. One source of information was the molar ratios between components, which had been published (Supplementary Figure 2a)[1,2]. In addition, we cloned the native RBS for each gene and measured their strengths using a fluorescent reporter (Supplementary Figure 2b and 20). This was done by cloning the 30bp region upstream of the ATG for the *prg-org* operon or -150 to +150 regions from the *inv-spa* operon into a plasmid containing a constitutive promoter and mRFP reporter and fluorescence was measured using flow cytometry (Methods). The expression levels were then approximately mimicked using synthetic RBSs and we decided to implement a minimum RBS strength for genes that we had evidence were expressed at low levels. When the *prg-org* operon was originally built the weakest RBS was relatively strong, but this yielded a functional refactored operon. To expand the number of expression levels we could set, a small RBS library was built and characterized in *Salmonella* prior to refactoring *inv-spa*. This was done by mutagenizing the BBa_B0030 RBS (Methods). *InvH* and *spaOs* were included at a later stage and the RBS Calculator was used to design their sequences (see Notes 1.D. and 1.E). The order of genes with the operons was also decided based on these data, with the most highly expressed genes appearing upstream.

1.B. The refactored prg-org operon in a p15a backbone under T7(T3) RNAP control*

The first refactored *prg-org* operon was designed and tested in the context of a low copy pSC101 plasmid under the control of the IPTG-inducible P_{tac} promoter and $lacI^{q}$ on the same plasmid (pMR_prgorgR1: *prg-org*^{R1}; Figure 2a, and Supplementary Figure 3a). We moved *prg-org* to a p15a backbone so that pSC101 could be used for *inv-spa* engineering (see next Notes). In addition, a controller was introduced to separate the regulation so that the IPTG-inducible system is carried on a separate plasmid and an attenuated version of T7 RNAP that has been engineered to only induce T3 RNAP promoters ($P_{tac\text{-}O2}$ driven T7*(T3): pMR_controller1) is used to induce *prg-org* [3]. This requires replacing the P_{tac} promoter with a T3 RNAP promoter of appropriate strength. The initial construct contained the P_{T3} promoter, obtained from previous work [3]. This yielded secretion, but even in the absence of inducer (however, when the controller was not present there was no secretion) (Supplementary Figure 3c). We then tested a series of promoters of different strength and found that one ($P_{T3.1}$) that enabled inducible secretion from the controller plasmid (Supplementary Figure 3d).

1.C. Library of chimeric native/refactored inv-spa operons

The first refactored *inv-spa* operon was synthesized and found to be non-functional. To determine which gene caused the loss of function, a series of operons were built that exchanged the RBS and codon sequence of each native gene with the synthetic RBS and sequence on the pMR_nativeinvspa plasmid, which encodes native *inv-spa* operon under P_{BAD} promoter control (Supplementary Figure 4). The chimeric plasmid was tested in the Δ*invF-spaS* strain (MR02). This demonstrated that function could be recovered when the native *spaO* sequence was included. Initially, we tried new genes synthesized with different codon usages, but this did not abrogate the problem. Finally, we built additional chimeras to look for internal sequences that needed to be retained (Note 1.D).

1.D. Identification and redesign of spaOs

Within the native *spaO* gene, the region spanning 456 to 610 nucleotides was found to be essential (Figure 2f). Possible alternative start codons were identified within this region, including codons that had been reported to serve as alternative start sites in prokaryotes [7,8]. In each case, we mutated the third nucleotide in the codon to disrupt the potential start site, but retain the amino acid encoded (Tables S4). This was done using the Δ*invF-spaS* knockout strain (MR02) containing the native *inv-spa* operon complemented on a plasmid (pMR_nativeinvspa). The ability to secrete the reporter was assayed for each of these mutations (Supplementary Figure 5). The result showed that *gtg* at 609 is the alternative start codon for tandem translation of *spaO* gene (Figure2h and 2i).

Since it was identified that both full length SpaO and smaller peptide SpaOs are necessary for secretion (Figure 2 and S5), *spaOs* was individually codon optimized and introduced into the refactored *inv-spa* operon. To control the expression level, a series of RBSs of different strength were designed b by the RBS Calculator online tool version (https://salis.psu.edu/software) using the RBS Calculator Design mode version 1.1, with organism option as *Salmonella enterica* subsp. *enterica* serovar Typhimurium str. LT2 (ACCTCCTTA) (Supplementary Table 5) (Methods) [9]. The location of *spaOs* was chosen to be between *invC* and *spaP* in the refactored operon in order to separate the gene from *spaO* yet separate from the highly expressed genes. The new *inv-spa* plasmids with the different RBSs were assayed for secretion in a Δ*invF-spaS* strain (MR02). Several strong RBSs were shown to recover secretion and we selected the strongest RBS (SBa_001710) from them.

1.E. Inclusion of invH in the refactored inv-spa operon

The function of InvH is to help localize the outer membrane ring component protein InvG [10]. Initially, we did not include the gene in the refactored *inv-spa* operon because it was previously shown to be non-essential [11] and it is encoded outside of the *inv-spa* operon in SPI-1. The gene was also found to be non-essential in our hands and it was not included in the first operons as a result. However, it was reported that the formation of needle complexes and invasion into epithelial cells were significantly reduced for *invH*-deficient Salmonella [11,12]. Therefore, we tested a codon-optimized version of *invH* to test for improved secretion. A series of RBSs for *invH* was designed by RBS Design mode version 1.1, with the organism set as *Salmonella enterica* subsp. *enterica* serovar Typhimurium str. LT2 (ACCTCCTTA) (Methods) [9]. This was tested in the context of the refactored *inv-spa* operon containing the *spaOs* gene. This refactored operon with *invH* was designated as *inv-spa*^{R2}.

1.F. Placement of the refactored inv-spa operon under T7(K1F) RNAP control*

The refactored *inv-spa* operon was initially tested using the arabinose-inducible P_{BAD} control where AraC is expressed from the same plasmid (previous Notes and Figures 2d-j). We wanted to select a RNAP that is orthogonal to T7*(T3) RNAP, used to control *prg-org*, so that the two operons could be induced independently. We had noticed that when the refactored *inv-spa* operon is highly induced, this can lead to cell leakage as detected by GroEL (not shown). Therefore, we decided to use the T7*(K1F) RNAP, which is orthogonal to T7*(T3) RNAP and weaker [3]. A controller was constructed where T7*(K1F) RNAP is under the control of the AHL-inducible P_{lux} system (Supplementary Figure 8b). The P_{BAD} promoter and inducible system were replaced with the P_{K1F} promoter in plasmid, pMR_invspaR3 (*inv-spa*^{R3}). This yielded a system where secretion could be induced in a Δ*invH-spaS* knockout (MR21) (Supplementary Figure 8).

1.G. Analysis of the refactored prg-org inv-spa operons in ΔprgH-orgCΔinvH-spaS

As an intermediate step, we analyzed the ability of the two refactored operons to complement secretion in a double knockout. This knockout retains the portions of SPI-1 external to these operons (Figure 1a). The two RNAPs were combined to build a single controller plasmid (pMR_controller3) where IPTG induces T7*(T3) RNAP and AHL induces T7*(K1F) RNAP (Supplementary Figure 17c and Methods). This controller was transformed with the plasmids containing refactored *prg-org* (pMR_prgorgR3: *prg-org*^{R3}) and *inv-spa* (pMR_invspaR3: *inv-spa*^{R3}) and tested for secretion in a *ΔprgH-orgCΔinvH-spaS* knockout (MR04; Figure 2b). Structure analysis by electron microscopy (EM) requires a non-flagellated strain of *Salmonella*. Therefore, we introduced an additional mutation into the Δ*prgH-orgC*Δ*invH-spaS* strain in order to disrupt the production of flagella Δ*flhDC* (MR06). Structures were isolated from the double knockout strain and characterized by EM (Figure 2b, right panel). Although all substructures were identified from refactored system, needle filament was distinctively short compared to native system.

Therefore, it was necessary to increase the expression of PrgI, a component for needle filament. To this end, ribozyme, RiboJ was added at the 5'-UTR of *prgI* gene in refactored operon, which also showed increased expression of PrgI/PrgJ (*prg-org*^{R4}; Figure 2c, Supplementary Figure 9b). The bacterial surface was analyzed obtained from the Δ*flhDC*Δ*prgH-orgC*Δ*invH-spaS* strain containing refactored *prg-org* operon tuned with RiboJ (*prg-org*^{R4}) together with refactored *inv-spa* operon (*inv-spa*^{R3}) (Supplementary Figure 9a). The needles were then purified, negative stained, and identified using EM (Supplementary Figure 9b). The refactored system produces needle complexes that are comparable in topology with the native operon and are consistent with functional T3SSs observed in previous work.

1.H. Search for SPI-1 regions outside of prgH-orgC and invH-iacP required for secretion

The two refactored *prg-org*^{R4}*inv-spa*^{R3} operons were able to complement activity in a double operon knockout (Figure 3b), but not when the complete SPI-1 was deleted. Prior to identifying *invR*, we hypothesized that there was a region in the deleted portion that was required for secretion. The possibilities included regulatory proteins (SprB, HilA, HilC, and HilD) and the *iagB* and *iacP* genes, as well a many effectors and chaperones (Supplementary Figure 10a). We made several deletion strains that also knocked out the region between *prgH* and *spaS* in addition to the two operons (Δ*prgH-orgC*Δ*invH-sipC*; Δ*prgH-orgC*Δ*invH-iacP;* Δ*invH-orgC*; Supplementary Figure 10a). This showed that the loss in secretion was due to the elimination of this region (Supplementary Figure 10b). This result indicates that the loss of activity is due to a factor in the genomic region between *sicP* and *hilD* (*sicP*, *sptP*, *iagB*, *hilA* and *hilD* including intergenic region between genes). Because both *sicP* and *sptP* are expressed from the reporter plasmid, this narrows the possibilities to *iagB*, *hilA*, and *hilD* together with intergenic region. However, the addition of IagB was unable to rescue the function of refactored system in ΔSPI-1 strain (Supplementary Figure 10c).

1.I. Deletion of invF and invE from the final refactored system

The *invF* and *invE* genes were known to be nonessential, but were included in the early versions of the refactored operons. This is because we wanted to maintain the regulatory feedback provided by InvF (for regulator/chaperone expression) and InvE had been shown to increase secretion of early substrates [13,14]. After building a functional refactored system, we tested for additional genes that could be deleted in the ΔSPI-1 background and these were found to be non-essential.

Supplementary Methods

Characterization of Salmonella enterica serovar Typhimurium LT2 DW01

Laboratory *S. typhimurium* was used as the wild type strain throughout the study. Genotying was performed by PCR analysis and sequencing of representative genes, including, *hisG*, *rpoS*, *rpsL*, and *xylR*. The full genome was also sequenced. Genomic DNA was isolated using gDNA purification kit (Promega, #A1125). Genome sequencing was performed on a Pacific Biosciences RS instrument (Broad Institute Genomics Platform). A total of 97,485 filtered subreads (441 Mb) were generated using 6 SMRT cells. BLASR software (Pacific Biosciences) was used for reference-guided assembly against LT2 (GenBank assembly GCA_000006945.1) and separately against SL1344 (GenBank Assembly GCF_000210855.2). Variant calling was then performed using Quiver (Pacific Biosystems). In sum, 236 variants were identified in the assembly based on LT2, and 2,715 variants were identified in the assembly based on SL1344. Based on the reduced variability indicates a closer evolutionary relationship, we focused on the LT2-based assembly. The genetic mutations are listed in Supplementary Table 1 in comparison with LT2. To reflect the different genotype, the strain is demarcated as DW01.

RNA-seq analysis

Strand specific RNA-seq was performed to determine the expression of the needle component proteins for the refactored and WT systems. Strains containing either system were cultured in IM (Methods) and the refactored system was induced with 10 µM IPTG and 10 µM AHL. Total RNA was isolated from each strain after 2 hrs of induction using PureLink RNA mini kit (Life Technologies #12183018A). Purified RNA samples were submitted for deep sequencing at MIT BioMicro Center (Cambridge, MA). Strand specific RNA-seq libraries were created by the BioMicro Center facility. The quality of RNA samples were assessed by fragment analyzer and nanodrop analysis then rRNA was removed by Ribozero kits (Bacteria; Illumina). After cDNA was synthesized by Illumina Truseq kits adding dUTP on second strand, the libraries were constructed by SPRIworks (Beckman Coulter) including amplification and barcoding. A reference genome was assembled by combining the laboratory wild type genomic sequence (Note 1.A.) with plasmids sequences. Reads were trimmed of barcodes and aligned to reference genome using BWA 0.7.10[15]. Strand-specific RPKM values were calculated using custom scripts which used the Bamtools API[16]. Read depth profiles were computed using the "mpileup –d 20000" function from the SAMtools suite[17]. For both computations, counts were added together from the replicates. Experimental error in RPKM errors was calculated using values obtained from biological replicates of strains.

Protein expression analysis of needle complex proteins

The needle complex protein expression levels were analyzed by western blot analysis from the refactored SPI-1 (MR30) in IM with 10 µM of IPTG and AHL. After 6 hrs, the bacterial cultures were harvested by centrifugation at 5,000 rpm for 15 mins. Each cell pellet was resuspended and a western blot was performed (Methods) using an anti-needle complex antibody as the primary antibody. For the comparison, we used non-flagellated *Salmonella typhimurium* SB905 carrying the plasmid pSB1418, which expresses transcriptional regulator *hilA* under the control of the P_{BAD} promoter. The purified needle complex from this strain at 6 hrs was used to show the expression levels (Supplementary Figure 13)[1]. The HilA overexpressed strain was used for the comparison because the WT strain had much lower levels of needle complex proteins expressed, making it hard to resolve their ratios (not shown).

The refactored SPI-1 in different strain backgrounds

The refactored system was transferred into additional *Salmonella* strains and tested for activity. Two strains were tested: *S. typhimurium* strain SL1344 (generous gift from Dr. Denise M. Monack, Professor of Stanford School of Medicine) and LT2 from ATCC (ATCC®700720™). First, ΔSPI-1 mutants were constructed for each strain by P22 phage transduction. Next, the reporter plasmid (pMR_reporter), the final plasmid-borne refactored version of SPI-1 (pMR_prgorgR5: *prg-org*^{R5} and pMR_invspaR4: *inv-spa*^{R4}), and controller plasmid (pMR_controller3) were sequentially introduced by electroporation.

Supplementary References

1. Marlovits, T. C. *et al.* Structural Insights into the Assembly of the Type III Secretion Needle Complex. *Science* **306,** 1040–1042 (2004).
2. Schraidt, O. & Marlovits, T. C. Three-dimensional model of Salmonella's needle complex at subnanometer resolution. *Science* **331,** 1192–1195 (2011).
3. Temme, K., Hill, R., Segall-Shapiro, T. H., Moser, F. & Voigt, C. A. Modular control of multiple pathways using engineered orthogonal T7 polymerases. *Nucleic Acids Res.* **40,** 8773–8781 (2012).
4. Kubori, T., Sukhan, A., Aizawa, S. I. & Galán, J. E. Molecular characterization and assembly of the needle complex of the Salmonella typhimurium type III protein secretion system. *Proc. Natl. Acad. Sci. U. S. A.* **97,** 10225–10230 (2000).
5. Widmaier, D. M. *et al.* Engineering the Salmonella type III secretion system to export spider silk monomers. *Mol. Syst. Biol.* **5,** 309 (2009).
6. Datsenko, K. A. & Wanner, B. L. One-step inactivation of chromosomal genes in Escherichia coli K-12 using PCR products. *Proc. Natl. Acad. Sci.* **97,** 6640–6645 (2000).
7. Ansong, C. *et al.* Experimental annotation of post-translational features and translated coding regions in the pathogen Salmonella Typhimurium. *BMC Genomics* **12,** 433 (2011).
8. Blattner, F. R. *et al.* The Complete Genome Sequence of Escherichia coli K-12. *Science* **277,** 1453–1462 (1997).
9. Salis, H. M. The ribosome binding site calculator. *Methods Enzymol.* **498,** 19–42 (2011).

10. Crago, A. M. & Koronakis, V. Salmonella InvG forms a ring-like multimer that requires the InvH lipoprotein for outer membrane localization. *Mol. Microbiol.* **30,** 47–56 (1998).

11. Altmeyer, R. M. *et al.* Cloning and molecular characterization of a gene involved in Salmonella adherence and invasion of cultured epithelial cells. *Mol. Microbiol.* **7,** 89–98 (1993).

12. Sukhan, A., Kubori, T., Wilson, J. & Galán, J. E. Genetic Analysis of Assembly of theSalmonella enterica Serovar Typhimurium Type III Secretion-Associated Needle Complex. *J. Bacteriol.* **183,** 1159–1167 (2001).

13. Eichelberg, K. & Galán, J. E. Differential regulation of Salmonella typhimurium type III secreted proteins by pathogenicity island 1 (SPI-1)-encoded transcriptional activators InvF and hilA. *Infect. Immun.* **67,** 4099–4105 (1999).

14. Kubori, T. & Galán, J. E. Salmonella type III secretion-associated protein InvE controls translocation of effector proteins into host cells. *J. Bacteriol.* **184,** 4699–4708 (2002).

15. Li, H. & Durbin, R. Fast and accurate short read alignment with Burrows-Wheeler transform. *Bioinforma. Oxf. Engl.* **25,** 1754–1760 (2009).

16. Barnett, D. W., Garrison, E. K., Quinlan, A. R., Strömberg, M. P. & Marth, G. T. BamTools: a C++ API and toolkit for analyzing and managing BAM files. *Bioinforma. Oxf. Engl.* **27,** 1691–1692 (2011).

17. Li, H. *et al.* The Sequence Alignment/Map format and SAMtools. *Bioinforma. Oxf. Engl.* **25,** 2078–2079 (2009).
